# Experimental approach to IGF-1 therapy in CCl_4_-induced acute liver damage in healthy controls and mice with partial IGF-1 deficiency

**DOI:** 10.1186/s12967-017-1198-4

**Published:** 2017-05-04

**Authors:** Luis A. Morales-Garza, Juan E. Puche, Gabriel A. Aguirre, Úrsula Muñoz, Mariano García-Magariño, Rocío G. De la Garza, Inma Castilla-Cortazar

**Affiliations:** 1 Escuela de Medicina, Tecnologico de Monterrey, Monterrey, Mexico; 2 Fundación de Investigación HM Hospitales, Madrid, Spain; 3 Department of Medical Physiology, School of Medicine, Universidad San Pablo-CEU, Madrid, Spain

**Keywords:** Acute liver damage, IGF-1, Oxidative damage, Hepatoprotection, Fibrogenesis

## Abstract

**Background:**

Cell necrosis, oxidative damage, and fibrogenesis are involved in cirrhosis development, a condition in which insulin-like growth factor 1 (IGF-1) levels are diminished. This study evaluates whether the exogenous administration of low doses of IGF-1 can induce hepatoprotection in acute carbon tetrachloride (CCl_4_)-induced liver damage compared to healthy controls (Wt *Igf* ^+/+^). Additionally, the impact of IGF-1 deficiency on a damaged liver was investigated in mice with a partial deficit of this hormone (Hz *Igf1* ^+/−^).

**Methods:**

Three groups of 25 ± 5-week-old healthy male mice (Wt *Igf* ^+/+^) were included in the protocol: untreated controls (Wt). Controls that received CCl_4_ (Wt + CCl_4_) and Wt + CCl_4_ were treated subcutaneously with IGF-1 (2 µg/100 g body weight/day) for 10 days (Wt + CCl_4_ + IGF1). In parallel, three IGF-1-deficient mice (Hz *Igf1* ^+/−^) groups were studied: untreated Hz, Hz + CCl_4_, and Hz + CCl_4_ + IGF-1. Microarray and real-time quantitative polymerase chain reaction (RT-qPCR) analyses, serum aminotransferases levels, liver histology, and malondialdehyde (MDA) levels were assessed at the end of the treatment in all groups. All data represent mean ± SEM.

**Results:**

An altered gene coding expression pattern for proteins of the extracellular matrix, fibrosis, and cellular protection were found, as compared to healthy controls, in which IGF-1 therapy normalized in the series including healthy mice. Liver histology showed that Wt + CCl_4_ + IGF1 mice had less oxidative damage, fibrosis, lymphocytic infiltrate, and cellular changes when compared to the Wt + CCl_4_. Moreover, there was a correlation between MDA levels and the histological damage score (Pearson’s r = 0.858). In the IGF-1-deficient mice series, similar findings were identified, denoting a much more vulnerable hepatic parenchyma.

**Conclusions:**

IGF1 treatment improved the biochemistry, histology, and genetic expression of pro-regenerative and cytoprotective factors in both series (healthy and IGF-1-deficient mice) with acute liver damage, suggesting that low doses of IGF-1, in acute liver damage, could be a feasible therapeutic option.

**Electronic supplementary material:**

The online version of this article (doi:10.1186/s12967-017-1198-4) contains supplementary material, which is available to authorized users.

## Background

Cell necrosis, inflammation, oxidative damage, hepatocellular regeneration, and fibrogenesis are processes that occur during the early stages of oxidative liver damage and lead to cirrhosis. Circulating levels of insulin-like growth factor-1 (IGF-1) are diminished in advanced cirrhosis [1, 2] due to the loss of biosynthetic mass. Moreover, it has been recently proposed that reduced hepatic production of insulin-like growth factor-1 (IGF-1) in ascitic liver disease could be one of the factors contributing to malnutrition in cirrhotic patients [3–5]. Moreover, our group has previously reported that, in an experimental cirrhosis model, low doses of IGF-1 improved nitrogen balance [3], jejunal sugar and amino-acid absorption [3, 4, 6, 7], osteopenia [8], testicular atrophy [9, 10], and somatostatinergic tone [11]. This treatment also induced additional hepatoprotective effects on the liver, including a reduction in lipid peroxidation, collagen content, and mechanisms of fibrogenesis with an improvement of histopathology, mitochondrial function, and antioxidant enzyme activities [5, 12–14].

As previously mentioned, serum IGF-1 levels are reduced in advanced liver cirrhosis; however, they are not applicable for acute liver oxidative damage. For this reason, the aim of the present work was to study whether low doses of IGF-1 resulted in hepatoprotection in carbon tetrachloride (CCl_4_)-induced acute liver damage in healthy controls. We have recently shown the deleterious effects of partial IGF-1 deficiency in the liver [15]. Thus, we have extended our studies to analyze the effect of low doses of IGF-1 on IGF-1-deficient mice (Hz, *Igf* ^+*/*−^) livers receiving acute CCl_4_-induced injury, in order to understand the impact and magnitude of such deficiency in terms of progression to liver cirrhosis.

## Methods

### Animals and experimental design

The experimental model was established and characterized as previously reported [16, 17]. Briefly, IGF-1 heterozygous mice were obtained by cross-breeding transgenic mice from lines 129SV and MF1^*Igf1*tm1Arge^. Animal genotype determination was performed by polymerase chain reaction (PCR) analysis (Applied Biosystems, 2720 Thermal Cycler, Spain). DNA was extracted from a piece of tail, and specific primers were used to identify both *Igf1* and *neo* genes (Extract-N-Amp TM Tissue PCR KIT Sigma, USA).

Animals were housed in cages in a room with a 12-h light/dark cycle and constant humidity (50–55%) and temperature (20–22 °C). Food (Tekland Global, 18% protein rodent diet, Harlan Laboratories, Spain) and water were given ad libitum. All experimental procedures were performed in compliance with The Guiding Principles for Research Involving Animals, and approved by the bioethical committees of our institutions (School of Medicine, Tecnologico de Monterrey, Monterrey, México, and CEU-San Pablo University, Madrid, Spain).

Three groups of 25 ± 5-week-old healthy male mice [wild-type (Wt), *Igf* ^+*/*+^] were included in the experimental protocol: untreated controls and wild-type mice (Wt *Igf* ^+*/*+^, n = 6); controls that received CCl_4_ (50 μl of CCl_4_) (n = 6) and Wt + CCl_4_ mice were subcutaneously treated with IGF-1 (2 μg/100 g/body weight/day) for 10 days (Wt + CCl4 + IGF-1, *Igf1* ^+*/*+^, n = 6). Another three groups of the same age, these with partial IGF-1 deficiency (Hz, *Igf1* ^+*/*−^), were studied in parallel: untreated Hz (*Igf1* ^+*/*−^, n = 6), Hz + CCl_4_ (n = 6), and Hz + CCl_4_ + IGF-1 (2 μg/100 g/body weight/day) for 10 days (n = 6). IGF-1 was provided by Chiron Corporation, USA.

On day 0, blood was drawn from the submandibular vein to determine IGF-1 serum levels before treatment. On day 11, mice were weighed, blood was obtained from the submandibular vein, and then mice sacrificed by cervical dislocation. The liver was carefully removed, weighed (Denver Instruments, Germany), and divided into two sections: the left lobe was stored in RNA later (Qiagen-Izasa, Spain) at −80 °C for microarray and real-time quantitative polymerase chain reaction (RT-qPCR) analyses, and the right lobe was used for histologic examination and malondialdehyde (MDA) assessment. Serum was stored at −20 °C.

### Histological study and semiquantitative liver damage and fibrosis scores

In liver sections stained with hematoxylin and eosin (H&E) and Masson’s trichrome, semiquantitative assessment of fibrosis and hepatic damage was blindly performed using a numerical scoring system, as used in previous works following validated models [18], based on the following criteria: (1) number and length of fibrous septa (0–2 points); (2) area of oxidative damage/necrosis (0–2 points); (3) lymphocyte infiltration as a marker of inflammation (0–1 points); (4) steatosis (0–1 points); and (5) cellular changes, such as misalignment of hepatocyte cords (0–1), aberrant nuclei (0–1), dead cells/cellular debris (0–1), and loss of the polyhedral shape of the hepatocytes (0–1); with a total partial score of 4 points (see Table 1).

**Table 1:** Body weights and liver weights (absolute and relative) of the three experimental groups of both series with and without IGF-1 deficiency (Hz and Wt, respectively)

	n	Vehicle	CCl_4_ (g)	CCl_4_ + IGF-1 (g)
Body weight (day 11)				
Healthy mice (Wt *igf* ^+*/*+^)	6	39.50 ± 1.64	39.40 ± 1.65	37.33 ± 1.26
Mice with IGF-1 deficiency (Hz *igf* ^+*/*−^ *)*	6	33.6 ± 1.07 *	34.2 ± 0.45*	32.17 ± 0.46*
Liver weight (day 11)				
Healthy mice (Wt *igf* ^+*/*+^)	6	2.01 ± 0.09	2.09 ± 0.14	1.93 ± 0.06
Mice with IGF-1 deficiency (Hz *igf* ^+*/*−^ *)*	6	1.78 ± 0.05*	1.73 ± 0.04**	1.87 ± 0.02
Relative liver weight (liver weight/body weight)				
Healthy mice (Wt *igf* ^+*/*+^)	6/6	5.27 ± 0.28	5.47 ± 0.29	5.21 ± 0.24
Mice with IGF-1 deficiency (Hz *igf* ^+*/*−^ *)*	6/6	5.60 ± 0.05	5.45 ± 0.20	6.05 ± 0.09*

* p < 0.05; ** p < 0.01 Hz vs corresponding control

All preparations were evaluated independently by three observers (double-blind), receiving a maximum score of 10 points. The arithmetical mean of the two scores was taken as the final score. This study was carried out using a light microscope (Leica, Switzerland). The slides were scanned using the Leica SCN400 (Leica Biosystems Pathology Imaging, Switzerland) and processed using Aperio ImageScope (v12.03.0.5056, Leica Biosystems Pathology Imaging).

### Serum IGF-1 and transaminases (AST and ALT) circulating levels

Serum IGF-1 levels were determined by an enzyme-linked immunosorbent assay (ELISA) commercial kit following specific commercial assay protocol instructions (Chiron Corporation, USA), read in a Varioskan spectrophotometer (Thermo Scientific, Spain), and interpreted using SkanIt software (Fisher Scientific, Spain).

Aspartate aminotransferase (AST) and alanine aminotransferase (ALT) were assessed by routine laboratory methods, using a Cobas-Hitachi autoanalyzer (Mannheim, Germany).

### MDA in liver homogenates

MDA was used as an index of lipid peroxidation, and was measured after heating samples at 45 °C for 60 min in an acidic medium. MDA was titrated by a colorimetric assay using 7.6 mM 1-methyl-2-phenylindole (modified from Gerard-Monnier) [19], which, after reacting with MDA, generates a stable chromophore that can be measured at 586 nm (Eppendorf Biophotometer plus, Eppendorf, Hamburg, Germany). Determinations were performed from liver homogenates immersed in Tris–hydrochloride (Tris–HCl) solution (1 g of liver tissue per 10 ml) centrifuged at 3000*g* during 10 min at 4 °C.

### Gene expression studies on the liver

#### Microarray analysis

Liver mRNA was isolated from animals belonging to each experimental group in accordance with the protocol outlined in the RNeasy Kit (Qiagen-Isaza, Spain). Technical procedures for microarray analysis, including quality control of mRNA, labeling, hybridization, and scanning of the arrays were performed according to standard operating procedures for Affymetrix protocols (GeneChipH Expression Analysis Manual, Affymetrix, USA). The mRNAs were profiled using Affymetrix HT MG-430 PM. The array signals were normalized using Robust Multichip Averages and batch-effects of the six replicates were corrected using ComBat. Differentially expressed genes between the six experimental groups were selected using FDR-corrected p values under 0.01 (p < 0.05).

#### Total RNA extraction, reverse transcription, and RT-qPCR

Hepatic lobules were cryopreserved in RNAlater (Qiagen-Izasa, Spain). The day RT-qPCR determinations were performed, hepatic samples were homogenized with TRIzol reagent (Invitrogen, UK) in TissueLyser LT (Qiagen-Izasa, Spain), and RNA was extracted and purified using the RNeasy Mini Kit (Qiagen, USA), including digestion with RNase-free DNase, following the manufacturer’s instructions. RNA quality was verified by A260/A280 ratio with a Nanodrop (Thermo Fisher Scientific Inc., USA), and fragment integrity and length were verified using a Bioanalyzer 2100 (Agilent Technologies Inc., USA). Purified RNA was then converted to cDNA using the RNA-to-DNA EcoDryTM Premix (Clonetech Labs, USA) for RT-qPCR assays. RT-qPCR assays were performed in a 3100 Avant Genetic Analyzer (Applied Biosystems Hispania, Spain). The thermal profile consisted of an initial 5-min melting step at 95 °C followed by 40 cycles at 95 °C for 10 s and 60 °C for 60 s.

The following specific Taqman^®^ probes for the selected genes were supplied by Applied Biosystems: *Igf1, Igf1r, Igf2, Asma, Adam11, Cat, Ccl2, Ccl5, Ccl12, Col1a1, Col1a2, Col4a3, Col4a4, Col4a5, Ctgf, Gpx1, Gpx8, Hif1a, Hsp90b1, Hspa4l, Hspa5, Hspa8, Hspa13, Hspb1, Ifng, Igfbp2, Igfbp3, Igfbp4, Igfbp5, Igfbp6, Igfbp7, Il1b, Mmp2, Mmp3, Mmp9, Pcna, Pdgfrb, Sp3, Tgfa, Tgfb1, Tgfb2, Timp, Tnfa, Vegfa, and Xiap*.

The relative mRNA levels of genes of interest were normalized to TBP expression using the simplified comparative threshold cycle delta, cycle threshold (CT) method [2^−(ΔCT gene of interest − ΔCT actin)^] [20].

### Statistical analysis

All data represent mean ± SEM. Statistical analysis was performed on SPSS 17 (IBM, USA). Significance was estimated by the U-Mann–Whitney test to compare non-parametric independent groups, or, when appropriate, by analysis of variance (ANOVA) to compare three parametric-dependent groups. The correlation between two parameters was analyzed by Spearman or Pearson’s tests. Differences were considered significant at a level of p < 0.05.

## Results

### Circulating levels of IGF-1 and body and liver weights

On day 0, significant differences of circulating levels of IGF-1 were found between healthy mice (Wt *Igf* ^+*/*+^) and Hz groups (Hz: 495.21 ± 63.41 vs. Wt: 653.19 ± 41.18 ng/ml, p < 0.01). Both groups of animals were divided into three experimental subgroups, as mentioned above.

On day 11, IGF-1-deficient animals (Hz) showed a significant reduction in body weight as compared to Wt groups (Table 1). Liver weights were also significantly reduced in the Hz and Hz + CCl_4_ groups as compared to their respective controls (Wt and Wt + CCl_4_). However, no significant differences in liver weight were observed between Wt + CCl_4_ and Hz + CCl_4_ groups treated with IGF-1 therapy (see Table 1).

When liver weight was referred to body weight (relative liver weight, mg of liver/g of body weight) no differences were found between groups, with the exception of the Hz + CCl_4_ group treated with IGF-1, which presented an increase in relative liver weight as compared to the Wt + CCl_4_ + IGF-1 group. Table 1 summarizes all of these data.

### Parameters of cytolysis and lipid peroxidation

**Fig. 1:**
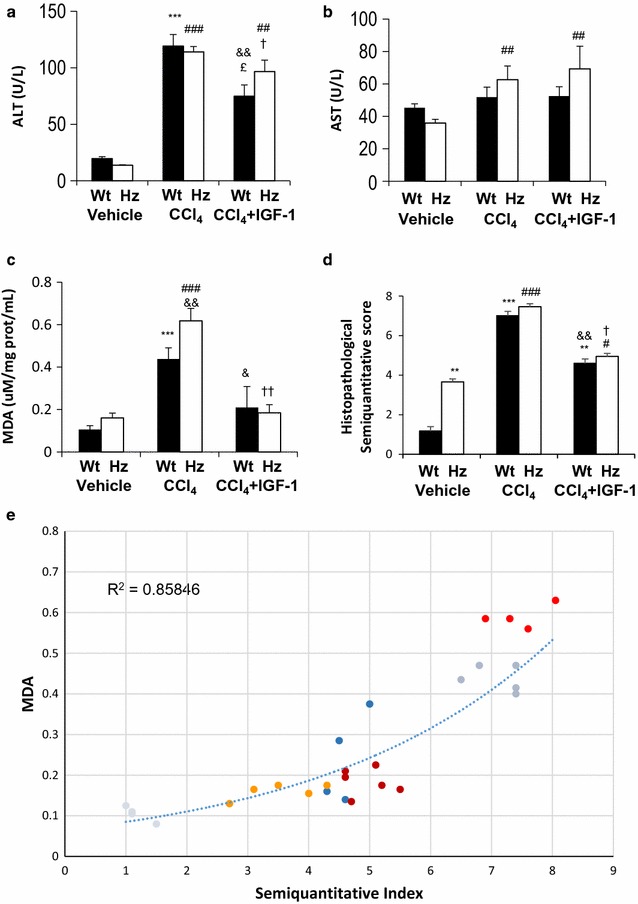
**a** Alanine transaminase levels. **b** Aspartate transaminase levels. **c** Malondialdehyde, as a marker of lipid peroxidation in liver homogenates. **d** Semiquantitative score of histological findings in the three experimental groups of both series (Wt and Hz animals); and **e** correlation between oxidative damage (MDA levels) and semiquantitative score of histological findings. **p < 0.01, ***p < 0.001 vs. Wt; ^#^p < 0.05, ^##^p < 0.01, ^###^p < 0.001 vs. Hz; ^&^p < 0.05, ^&&^p < 0.01 vs. Wt + CCl_4_; ^†^p < 0.05, ^††^p < 0.01 vs. Hz + CCl_4_; ^£^p < 0.05 vs. Hz + CCl_4_ + IGF-1

On day 11, ALT levels were found to be significantly increased in both the Wt + CCl_4_ and Hz + CCl_4_ groups as compared to the Wt and Hz groups (Fig. 1a). ALT levels were also higher in the Hz + CCl_4_ + IGF-1 group as compared to the Wt + CCl4 +IGF-1 group (p < 0.01). AST levels were only increased in the Hz group receiving CCl_4_. No effect of IGF-1 was found for this parameter (Fig. 1b).

In both experimental groups, the hepatic levels of the lipid peroxidation products (estimated as nmol of MDA/g of tissue) were significantly increased in mice receiving CCl_4_, whereas IGF-1 dramatically reduced them to similar values to those found in controls (Fig. 1c). An increase in this lipid peroxidation marker was also found in Hz mice compared to that found in Wt groups, suggesting that the single IGF-1 deficiency renders the liver more vulnerable to oxidative damage. IGF-1 therapy was useful in both groups (Wt + CCl_4_ + IGF-1 and Hz + CCl_4_ + IGF-1), as shown in Fig. 1c.

### Liver histology and semiquantitative score

**Fig. 2:**
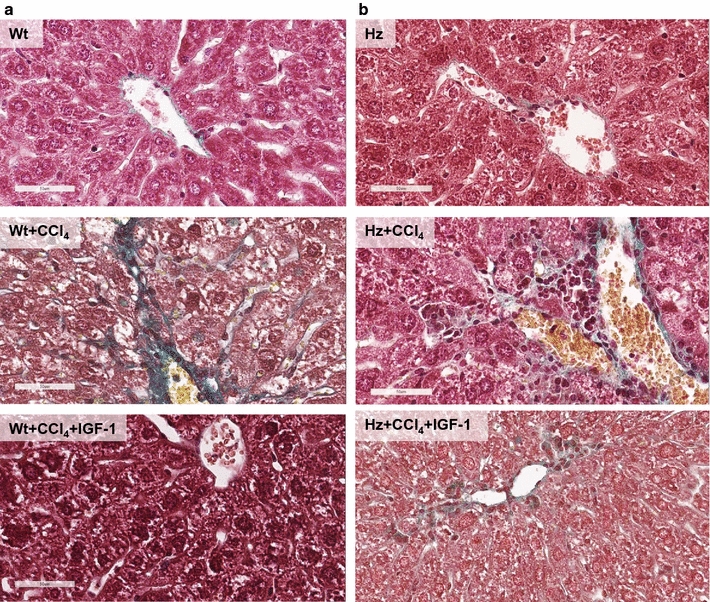
**a** Liver histological study (Masson’s trichrome, ×40) in the three experimental groups of healthy mice (Wt *Igf* ^+*/*+^). **b** Liver histological study (Masson’s trichrome, ×40) in the three experimental groups of IGF-1-deficient mice (Hz *Igf* ^+*/*−^), with partial IGF-1 deficiency

Figure 2a shows relevant findings in the three experimental groups of healthy mice (Wt). The histopathological score was significantly lower in the Wt + CCl_4_ + IGF-1 group than in the Wt + CCl_4_ group (Wt + CCl_4_ + IGF-1: 4.68 ± 0.08 vs. Wt + CCl_4_: 7.01 ± 0.16 arbitrary units, p < 0.01) (Fig. 1d; Table 2).

**Table 2:** Results of histopathological findings estimated for the total semiquantitative score

Experimental group	Oxidative damage (0–2)	Lymphocytic infiltrate (0–1)	Fibrosis (0–2)	Steatosis (0–1)	Cellular changes (0–4)	Total score (0–10)
Wt	0.08 ± 0.03	0.05 ± 0.03	0.10 ± 0.04	0.10 ± 0.00	0.68 ± 0.13	1.00 ± 0.10
Wt + CCl_4_	1.30 ± 0.09*	0.53 ± 0.04*	1.20 ± 0.04*	0.73 ± 0.04*	3.26 ± 0.10*	7.01 ± 0.16**
Wt + CCl_4_ + IGF-1	0.75 ± 0.03*^&^	0.65 ± 0.10*	0.38 ± 0.05*^&^	0.73 ± 0.05*	2.18 ± 0.10*^&^	4.68 ± 0.08*^&^
Hz	0.38 ± 0.04*	0.16 ± 0.02	0.20 ± 0.03	0.14 ± 0.02	2.78 ± 0.14*	3.66 ± 0.29*
Hz + CCl_4_	1.60 ± 0.04^#£^	0.78 ± 0.03^#£^	1.40 ± 0.04^#^	0.73 ± 0.03^#^	3.50 ± 0.21^#^	8.00 ± 0.17^##£^
Hz + CCl_4_ + IGF-1	0.73 ± 0.03^##$$^	0.60 ± 0.05^##$^	0.35 ± 0.04^#$$^	0.78 ± 0.03^##^	2.52 ± 0.19^$$^	4.98 ± 0.25^#$^

* p < 0.05 vs Wt; ** p < 0.01 vs Wt; ^&^p < 0.05 vs Wt + CCl4; ^#^p < 0.05 vs Hz; ^##^p < 0.01 vs Hz; ^$^p < 0.05, ^$$^p < 0.01 vs Hz + CCl4; ^£^p < 0.05 vs Wt + CCl4

Figure 2b corresponds to histological findings from the three IGF-1-deficient groups (Hz). The histopathological score was also found to be reduced in IGF-1-treated mice (Hz + CCl_4_ + IGF-1) as compared to untreated mice receiving CCl_4_ (Hz + CCl_4_ + IGF-1: 4.98 ± 0.25 vs Hz + CCl_4_: 8.00 ± 0.17, p < 0.01). Data are summarized in Table 2.

Emphasis must be placed livers from IGF-1-deficient mice, which show a greater vulnerability to CCl_4_-induced damage, expressing higher rates of lymphocytic infiltrates (Wt + CCl_4_, 0.53 ± 0.04 vs. Hz + CCl_4_, 0.78 ± 0.03 p < 0.05) and oxidative damage (Wt + CCl_4_, 1.30 ± 0.09 vs. Hz + CCl_4_, 1.60 ± 0.04 p < 0.05) (see Table 2).

Interestingly, a direct and significant correlation was found between MDA levels and the semiquantitative scores of histopathological damage (Pearson’s r = 0.858; Fig. 1e).

### Gene expression studies of the liver

In light of these results, a study of hepatic gene expression was performed. Microarray analysis revealed 211 genes that were either over-expressed or under-expressed in IGF-1-deficient animals as compared to controls (fold-change over ± 1.5) and Hz mice treated with IGF-1 (see Additional file 1: Table S1).

Among genes with an altered expression, we focused on those coding for: (1) inflammatory, fibrogenic, or regenerative factors, and antioxidant enzymes; (2) proteins of the extracellular matrix (ECM) and its regulators; and (3) cytoprotective molecules involved in survival and mitochondrial protection.

RT-qPCR was subsequently performed to confirm changes depicted by the microarray over ±1.5 fold-change.

A significant increase in *Igf1r* was confirmed in the Hz + CCl_4_ group (1.712 ± 0.282; p < 0.05 vs. control), which was restored by IGF-1 therapy (Hz + CCl_4_ + IGF-1 = 0.817 ± 0.147).

**Fig. 3:**
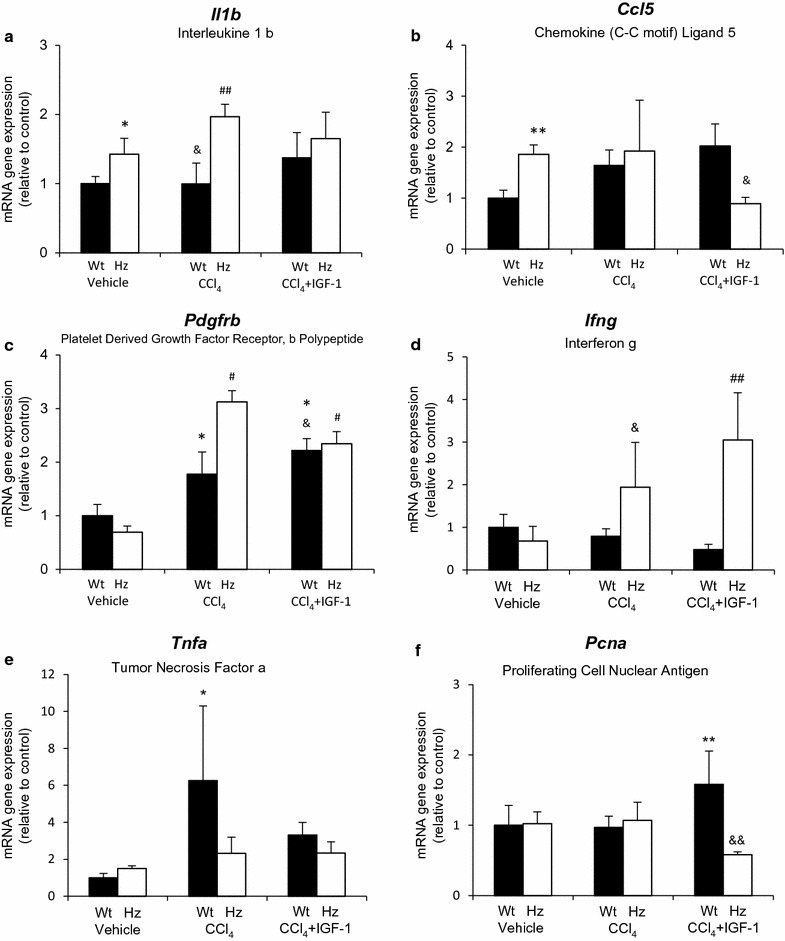
Data from RT-qPCR for genes expressing (mRNA) encoding growth factors involved in inflammation (**a** *Il1b*, **b** *Ccl5*, **c** *Pdgfr*, **d** *Ifng*, **e** *Tnfa*) and cell proliferation (**f** *Pcna*). *p < 0.05, **p < 0.01 vs controls (Wt); ^#^p < 0.05, ^##^p < 0.01 vs Hz; ^&^p < 0.05, ^&&^p < 0.01 vs corresponding group of the other series (i.e., Wt + CCl_4_ vs Hz + CCl_4_, or Wt + CCl_4_ + IGF-1 vs Hz + CCl_4_ + IGF-1)

**Fig. 4:**
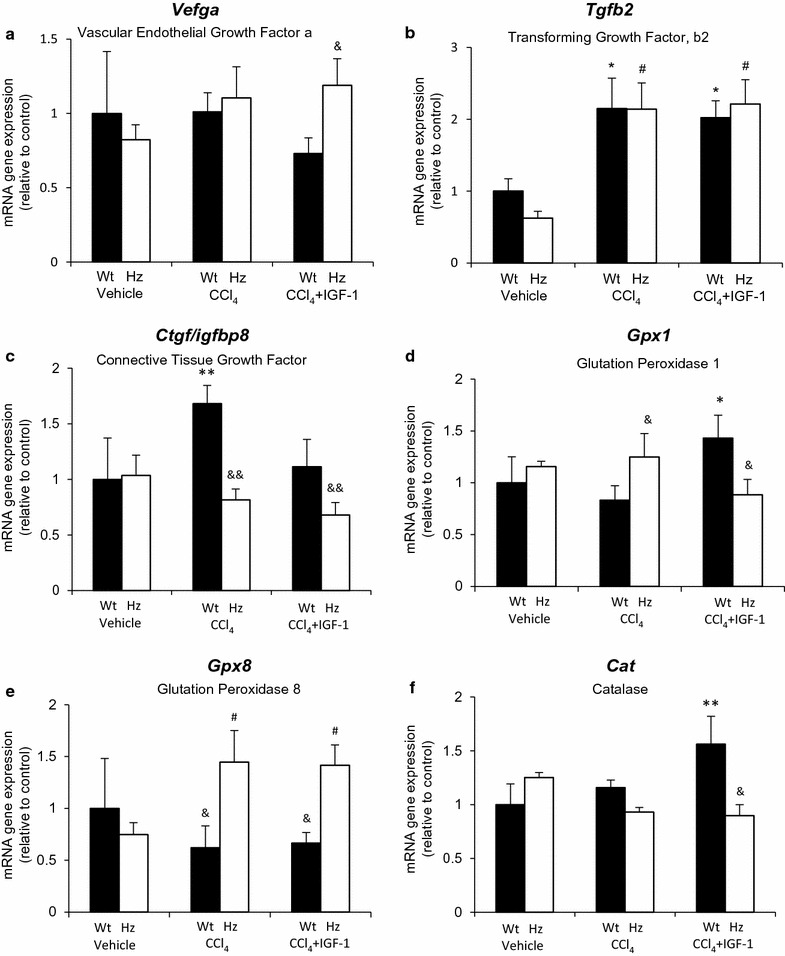
RT-qPCR results for gene expression (mRNA) involved in angiogenesis (**a** *Vegfa*), fibrogenesis (**b** *Tgfb2*, **c** *Ctgf*), and antioxidants enzymes (**d** *Gpx1*, **e** *Gpx8*, and **f** *Cat*). *p < 0.05, **p < 0.01 vs controls (Wt); ^#^p < 0.05, ^##^p < 0.01 vs Hz; ^&^p < 0.05, ^&&^p < 0.01 vs corresponding group of the other series (i.e., Wt + CCl_4_ vs Hz + CCl_4_, or Wt + CCl_4_ + IGF-1 vs Hz + CCl_4_ + IGF-1)

Figure 3 summarizes RT-qPCR results from genes that encode growth factors involved in inflammation (*Il1b*, *Ccl5*, *Pdgfrb*, *Ifng*, *Tnfa*) and cell proliferation (*Pcna*). Additionally, Fig. 4 shows genes related to angiogenesis (*Vegf*), fibrogenesis (*Tgfb2*, *Ctgf*), and antioxidative enzymes (*Gpx1*, *Gpx8*, and *Cat*). In accordance with previous data [19], IGF-1 deficiency increased gene expression of pro-inflammatory factors, such as *Ccl5* and *Il1b*.

Hz animals responded differently than healthy ones (Wt) after CCl_4_ injury or IGF-1 therapy (*Ifng*, *Pcna*, *Tnfa*, *Vegf*, *Ctgf*, *Gpx1*, *Gpx8*, *Cat*).

In addition, *Pcna* gene expression was only increased in Wt + CCl_4_ + IGF-1 mice (p < 0.01 vs. other groups, including Hz + CCl_4_ + IGF-1), representing an increment in cellular proliferation. *Tgfb2* expression was increased in all groups receiving CCl_4_ insult as compared to their corresponding controls (Wt or Hz). IGF-1 treatment did not modulate its expression in the Wt + CCl_4_ nor Hz + CCl_4_ groups.

*Ifng* expression was only augmented in the Hz + CCl_4_ group, particularly after IGF-1 treatment. *Tnfa* expression showed a significant increase in Wt + CCl_4_ animals, which is indicative of cell death. Surprisingly, animals with IGF-1 deficiency lacked such a physiological response.

With regard to genes encoding antioxidant enzymes, IGF-1 therapy increased the expression of catalase (CAT) and glutathione peroxidase (GPX1) in Wt + CCl_4_ mice. However, it had no effect on *Gpx8* gene expression.

**Fig. 5:**
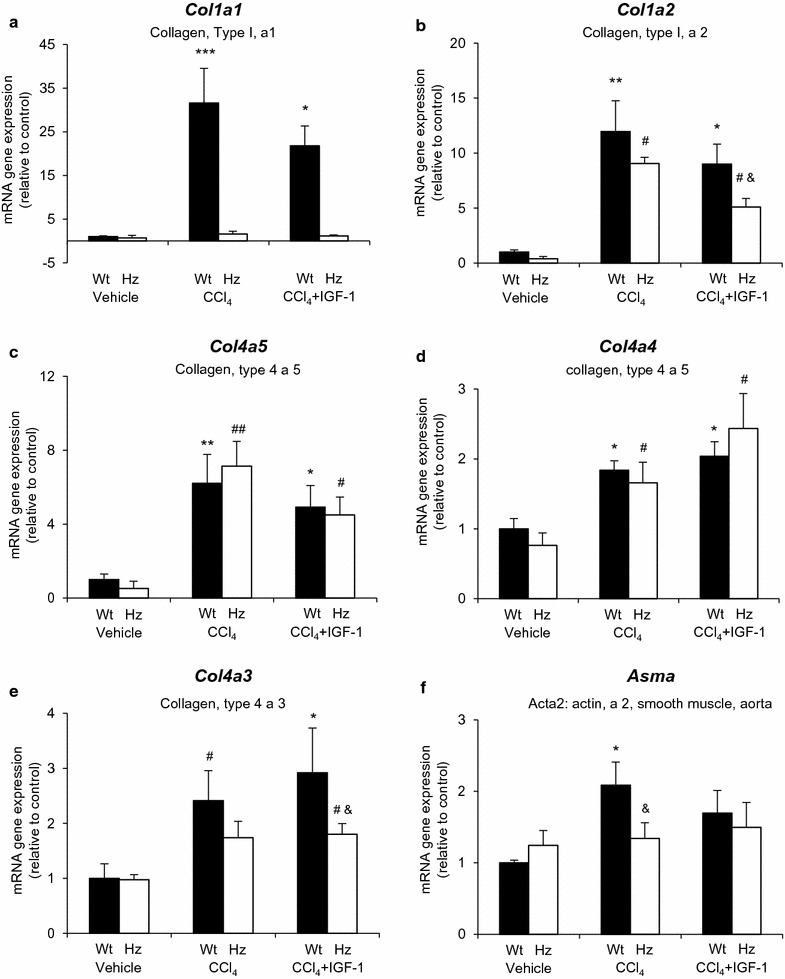
Gene expression (mRNA) measured by RT-qPCR coding for collagens (**a** *Col1a1*, **b** *Col1a2*, **c** *Col4a5*, **d** *Col4a4*, **e** *Col4a3*), and aorta smooth muscle actin a2 (**f** *Asma*). *p < 0.05, **p < 0.01, ***p < 0.001 all groups vs controls (Wt + vehicle); ^#^p < 0.05, ^##^p < 0.01 Hz + CCl_4_ or Hz + CCl_4_ + IGF-1 vs corresponding control group (Hz + vehicle); ^&^p < 0.05 between Wt + CCl_4_ + IGF-1 vs Hz + CCl_4_ + IGF-1 or Wt + CCl_4_ vs Hz + CCl_4_

**Fig. 6:**
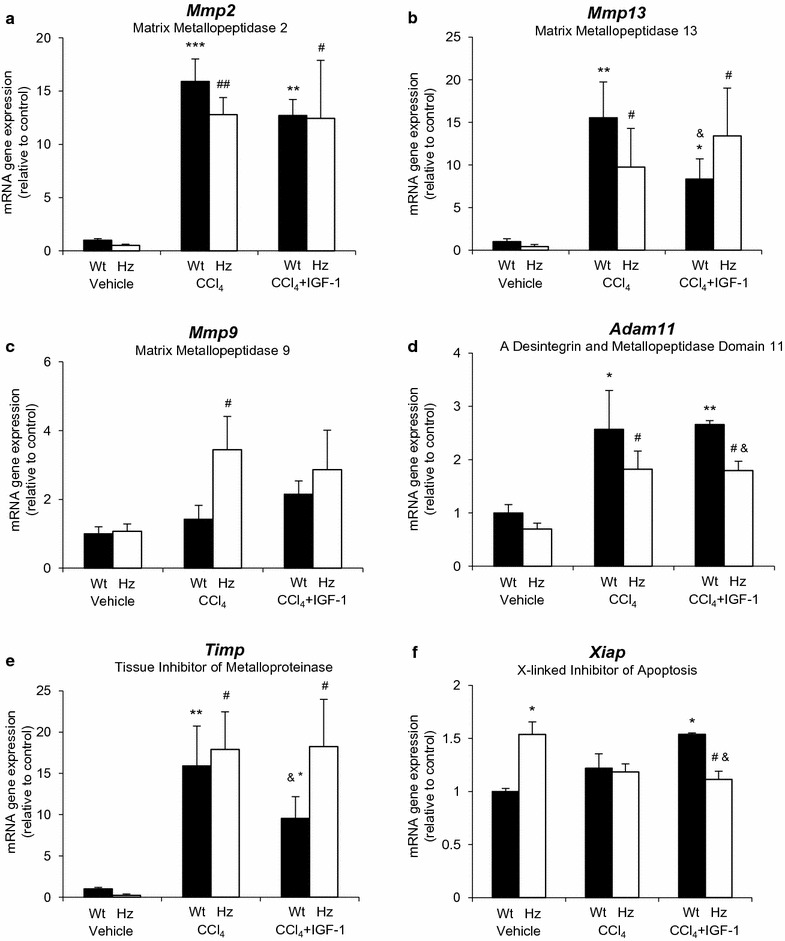
RT-qPCR of gene expression (mRNA) that encodes proteases (**a** MMP2, **b** MMP13, **c** MMP9 and **d** a desintegrin and metallopeptidase—*Adam11*) as well as its inhibitor (**e** *Timp1*), and the inhibitor of apoptosis (**f** *Xiap*). *p < 0.05, **p < 0.01, ***p < 0.001 all groups vs controls (Wt + vehicle); ^#^p < 0.05, ^##^p < 0.01 Hz + CCl_4_ or Hz + CCl_4_ + IGF-1 vs corresponding control group (Hz + vehicle); ^&^p < 0.05 between Wt + CCl_4_ + IGF-1 vs Hz + CCl_4_ + IGF-1 or Wt + CCl_4_ vs Hz + CCl_4_

Figure 5 includes results from genes coding for several types of collagen, its regulators [metalloproteinases (MMPs) and a desintegrin and metalloproteinase (ADAM)], and their inhibitors (TIMPs) (Fig. 6). Additionally, we have included gene expression for actin-alfa-2 (ASMA) as a marker of stellate cell transformation to myofibroblasts (Fig. 5).

Surprisingly, Hz mice did not respond to CCl_4_ injury by expressing *Col1a1*, while healthy mice (Wt + CCl_4_) expressed it in significant increments. However, the expression of the other collagen types depicted a reduction following IGF-1 therapy in Wt + CCl_4_ mice, with the only exception of *Col4a3*.

Regarding the gene expression of MMPs (*Mmp2*, *Mmp13*, *Mmp9*, and *Adam11*), an expected increase after CCl_4_ injury was observed and accompanied by a reduction after IGF-1 therapy that did not reach statistical significance.

Finally, the expression of *Asma* (actin-alfa-2) was significantly increased in healthy mice receiving CCl_4_, but not in IGF-1-deficient animals, which revealed a significant reduction when compared to the Wt + CCl_4_ group (Fig. 5).

**Fig. 7:**
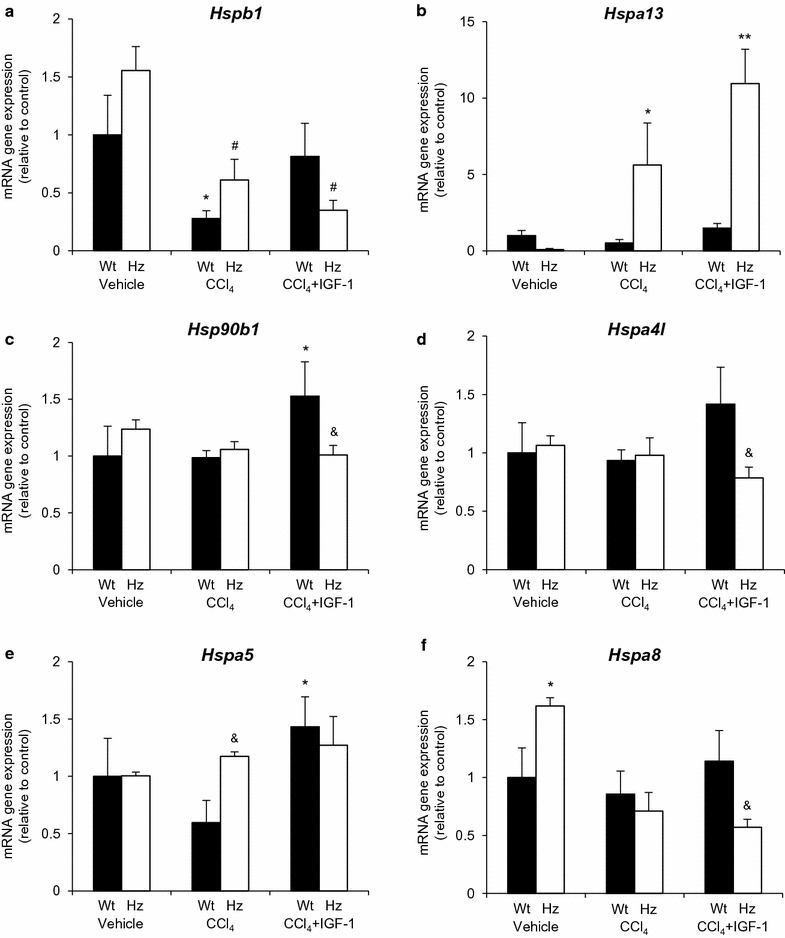
Gene expression (mRNA) measured by RT-qPCR coding for heat shock proteins (HSPs) involved in cytoprotective activities. *p < 0.05, **p < 0.01, ***p < 0.001 all groups vs controls (Wt + vehicle); ^#^p < 0.05, ^##^p < 0.01 Hz + CCl_4_ or Hz + CCl_4_ + IGF-1 vs corresponding control group (Hz + vehicle); ^&^p < 0.05 between Wt + CCl_4_ + IGF-1 vs Hz + CCl_4_ + IGF-1 or Wt + CCl_4_ vs Hz + CCl_4_

Figure 7 summarizes the gene expression of chaperones, heat shock proteins, proteins involved in cytoprotective activities, and *Xiap* (X-linked inhibitor of apoptosis) (Fig. 6). Results indicated that *Hsp* gene expression (*Hspb1*) was significantly diminished in both groups receiving CCl_4_ as compared with their corresponding controls (Wt or Hz). Expression of *Hspa13* was also significantly increased by IGF-1 therapy, as well as *Hsp90b1*, *Hspa41*, and *Hspa5* in Wt + CCl_4_ + IGF-1 mice.

IGF-1 deficiency significantly increased the expression of *Xiap* (Fig. 6) and *Hspa8* (Fig. 7), while the expression of both of them was found to be reduced by IGF-1 therapy (Hz + CCl_4_ + IGF-1). Even though the expression of *Hspa8* was not changed in the three experimental groups of Wt animals, IGF-1 therapy significantly increased the expression of *Xiap*.

## Discussion

Results in this paper demonstrate that low doses of IGF-1, for short periods of time, induce hepatoprotective, antioxidant, and antifibrogenic effects in animals with acute liver damage, in accordance with previous results from experimental cirrhosis [5, 12, 13]. These findings suggest that low-dose-IGF-1 therapy could be an effective strategy to avoid the progression to cirrhotic hepatic disease in early stages of liver damage.

The histopathological damage induced by only three doses of CCl_4_, and the rapid recovery of the hepatic architecture in only 10 days of the experimental period in which the IGF-1 treatment was simultaneously administered with CCl_4_ is worthy of special mention. This can be seen in Fig. 2.

Healthy mice within the experimental model (Wt, *Igf1* ^+*/*+^) appeared to be suitable for testing the potential of therapeutic strategies to attenuate acute hepatic damage. Histopathological changes (lymphocytic infiltration, fibrosis, oxidative damage, steatosis, and serious cellular changes; see Table 2) were associated with a significant increase in hepatic lipid peroxidation levels (see Fig. 1c), which were restored to normal values following IGF-1 therapy.

Groups including healthy mice (not harboring *Igf1* mutation Wt, *Igf1* ^+*/*+^) displayed a predictable genetic expression response to CCl_4_-induced oxidative damage, which was also the case for the IGF-1-treated mice. Oxidative insult increased the expression of all those genes encoding collagen proteins (see Fig. 5a–e), all of which were reduced by IGF-1 treatment, with the exception of collagens 3, 4α, and 4. Similarly, the gene coding expression for actin α2, as a marker for stellate cell differentiation to myofibroblasts, which produce collagens and proteoglycans during the fibrogenic process [21], was increased in Wt + CCl_4_ and reduced (p = ns vs. controls) in Wt + CCl_4_ receiving IGF-1 therapy.

On the other hand, TNF-α gene expression was significantly augmented in Wt + CCl_4_, showing a normal inflammatory response suggesting a better hepatic regeneration capability [22]. Additionally, IGF-1 therapy increased the expression of proliferation cell nuclear antigen (PCNA) in the Wt + CCl_4_ + IGF-1 group, suggesting an efficient stimulus for liver regeneration.

Gene expression assays revealed both antifibrogenic and fibrolytic properties for IGF-1 therapy, modulating the expression of collagen and MMP genes, as well as the inhibitor of MMPs (TIMP). IGF-1 therapy also reduced lipid peroxidation and increased the expression of genes coding for the antioxidant enzyme CAT (Fig. 4f).

The depletion circulating levels of IGF-1 in advanced liver cirrhosis is well documented; however, this does not occur in acute damage. Thus, this work aimed to link the consequences of chronically low levels of IGF-1 and acute liver damage, as several conditions resemble such a deficiency (metabolic syndrome [23], aging [24]…), which could render individuals more sensible to liver aggression. With this objective, we used a recently characterized model of haploinsufficient, heterozygous (*Igf1* ^+*/*−^) mice, which show partial IGF-1 deficiency.

The IGF-1 deficiency (Hz group) was associated with histological alteration with loss of hepatocyte cord alignment, apparent disruption of cell polarity, aberrant nuclei, and abundant cytosolic vacuolization. All of these are in accordance with previous results [15], where altered gene coding expression patterns were described for cytoskeleton proteins, as well as genes related to hepatocyte polarity, cell junctions, and ECM proteins. In fact, in the present work, the histopathological scores for histological damage and lipid peroxidation levels were significantly higher in the Hz group compared to controls (Wt group) (Fig. 1c, d).

In this context, mice with partial IGF-1 deficiency (Hz *Igf* ±), which received exogenous CCl_4_ insult (Hz + CCl_4_), bared a marked inflammatory response and oxidative damage (Figs. 1c, d , 3a–d; Table 2), whereas the expression of gene coding proteins involved in the ECM was paradoxical: hypo-expression of *col1a1*, hyper-expression of *Timp* (fibrolysis inhibitor), and reduction of *Asma* (alfa2-actin), which is a marker for stellate cell transformation into myofibroblasts [21]. Therefore, the genetic response of ECM proteins after CCl_4_ injury differs among animals with and without IGF-1 deficiency (Hz and Wt, respectively). In particular, the expression of collagen-alfa type-1, one of the main components of hepatic ECM, is virtually absent, even after the sole CCl_4_ administration in IGF-1-deficient mice.

Surprisingly, collagen deposition was similar in both groups (Wt and HZ) receiving CCl_4_ (Table 2), probably because in Hz groups, the fibrolytic mechanisms were also reduced by TIMPs (Fig. 7e).

Another potentially relevant finding could be the role of connective tissue growth factor (CTGF/IGFBP8) in the normal or pathological establishment of the ECM. This protein is a multi-modular molecule that binds and activates other factors, such as TGF-β and VEGF, and also possesses a domain for IGF-1. It is recognized as an ECM “organizer” [22]. Our results showed that after CCl_4_ insults in healthy mice, *Ctgf* expression increased significantly compared to controls, and that IGF-1 therapy downregulated its expression to normal values (Fig. 4c). However, IGF-1-deficient mice expressed low levels of *Ctgf* under all tested scenarios (Hz + CCl_4_, Hz + CCl_4_ + IGF-1).

The gene expression of cytoprotective heat shock proteins (HSPs) (Fig. 7), appeared to be acting as the protective mediator for IGF-1 activities, as previously reported in brain samples [20]. It is well known that HSPs act as sensors of cellular redox changes and contribute to the repair and clearance of damaged proteins [21]. Some of them, such as *Hsp27*, *Hsp70* and *Hsp90*, play an important role in inhibiting apoptosis and inflammation [21].

In summary, this work supplies novel evidence suggesting low-dose-IGF-1 therapy as being potentially beneficial in avoiding the progression of early stages of liver damage to more chronic and deleterious conditions. In the early stages of hepatic disease, IGF-1 treatment induces cell regeneration and cytoprotection, and is also effective as an anti-inflammatory and antioxidant agent, resulting in a concomitant reduction in fibrosis (both fibrogenesis and fibrolysis).

## Conclusions

To conclude, firstly, low doses of IGF-1 induce hepatoprotective, antioxidant, and antifibrogenic effects in animals after acute liver damage. IGF-1 also modulates the expression of collagen and MMP genes in animals with acute liver damage induced by CCl_4_, including antifibrogenic and fibrolytic mechanisms. Secondly, IGF-1 exogenous administration could be a strategic therapy in the early stages of liver damage, avoiding the progression to cirrhosis. Lastly, IGF-1 deficiency, present in liver cirrhosis, represents a negative factor for the magnitude and proportion of liver damage, since the livers from IGF-1-deficient mice (Hz *Igf1* ^+/−^) show a clear vulnerability to oxidative damage and inflammation.

## Additional file

**Additional file 1: Table S1.** Additional table.

## Abbreviations

ALT: alanine aminotransferase
AST: aspartate aminotransferase
GSHPx: glutathione peroxidase
CCl_4_: carbon tetrachloride
HSP: heat shock proteins
IGF-1: insulin-like growth factor-1
MDA: malondialdehyde
ns: not significant
Hz: heterozygous group
*Igf*^+*/*−^: mice with partial IGF-1 deficiency
Hz + CCl_4_ + IGF-1: heterozygous (Hz, *Igf* ^+*/*−^) receiving CCl_4_ and treated with low doses of IGF-1
MMP: matrix metalloprotease
RT-qPCR: real time quantitative polymerase chain reaction
SEM: standard error of mean
Wt: control group (wild-type, *Igf* ^+*/*+^)
EMC: extracellular matrix
HSC: hepatic stellate cells
IGFBPs: IGF binding proteins
IGF-1: insulin-like growth factor-1
IGF-1R: IGF-1 receptor
rhIGF-1: recombinant human insulin-like growth factor
TGF-β1: transforming growth factor-β1
*Xiap*: X-linked inhibitor of apoptosis
