# Supplementary material for: Experimental approach to IGF-1 therapy in CCl4-induced acute liver damage in healthy controls and mice with partial IGF-1 deficiency
Source: J Transl Med. 2017 May 4;15:96. doi: 10.1186/s12967-017-1198-4 (PMC5418730; doi:10.1186/s12967-017-1198-4)
Supplement: Supplementary file 1 — Additional file 1: Table S1. Additional table. [file 12967_2017_1198_MOESM1_ESM.docx]

|  |  | WT vs. WT+CCl_4_ | | WT vs. WT+CCl_4_+IGF-1 | | Hz vs. Hz+CCl_4_ | | Hz vs. Hz+CCl_4_+IGF-1 | | WT vs. Hz | |
| --- | --- | --- | --- | --- | --- | --- | --- | --- | --- | --- | --- |
| **Gene** | Gene name | p-value | Fold-Change | p-value | Fold-Change | p-value | Fold-Change | p-value | Fold-Change | p-value | Fold-Change |
| ***Vegfc*** | vascular endothelial growth factor C | 0.0224182 | -1.50549 | 0.844689 | 1.02779 | 0.0559777 | -1.37267 | 0.377018 | -1.13633 | 0.14852 | 1.24867 |
| ***Pdgfa*** | platelet derived growth factor, alpha | 0.0166151 | -1.51196 | 0.359727 | -1.13268 | 0.092882 | -1.28514 | 0.0221109 | -1.46964 | 0.275813 | 1.16257 |
| ***Pcna*** | proliferating cell nuclear antigen | 0.00026482 | -1.51573 | 0.0186597 | -1.19046 | 0.0126189 | -1.21115 | 0.00029746 | -1.5026 | 0.105149 | 1.10958 |
| ***Aldh1a1*** | aldehyde dehydrogenase family 1, subfamily A1 | 2.23E-06 | -1.5209 | 0.583352 | -1.01398 | 0.16922 | -1.03815 | 0.00024032 | 1.20444 | 0.00022446 | -1.20722 |
| ***Aldh1l1*** | aldehyde dehydrogenase 1 family, member L1 | 0.0766236 | -1.52158 | 0.191584 | -1.33539 | 0.223808 | -1.30552 | 0.964402 | -1.00919 | 0.508152 | -1.14828 |
| ***Dnajc21*** | DnaJ (Hsp40) homolog, subfamily C, member 21 | 0.037594 | -1.5273 | 0.213347 | -1.24823 | 0.252075 | -1.22366 | 0.0195579 | -1.65436 | 0.151985 | 1.29867 |
| ***Il2rg*** | interleukin 2 receptor, gamma chain | 0.0266572 | -1.53505 | 0.995773 | -1.00081 | 0.250541 | -1.20526 | 0.479431 | -1.11704 | 0.388721 | -1.14613 |
| ***Gpt*** | glutamic pyruvic transaminase, soluble | 0.00012789 | -1.53511 | 1.90E-05 | -1.81955 | 0.33295 | 1.05327 | 0.0197934 | -1.16805 | 0.00905237 | -1.20555 |
| ***Ldhb*** | lactate dehydrogenase B | 1.76E-05 | -1.53767 | 0.776368 | 1.01045 | 0.0502068 | -1.08922 | 0.0770936 | -1.07737 | 0.22556 | -1.04836 |
| ***Irf9*** | interferon regulatory factor 9 | 8.78E-05 | -1.54277 | 0.00100508 | -1.32011 | 0.383092 | -1.04487 | 9.79E-05 | -1.53001 | 0.00324108 | 1.24661 |
| ***Cd72*** | CD72 antigen | 0.00015637 | -1.54863 | 0.042332 | -1.14341 | 0.00063322 | -1.40319 | 0.898912 | 1.00693 | 0.178519 | -1.08267 |
| ***Aifm1*** | apoptosis-inducing factor, mitochondrion-associated 1 | 0.00022966 | -1.54875 | 0.00073536 | -1.42336 | 0.00518403 | -1.27051 | 0.00040045 | -1.4848 | 0.0501601 | 1.1464 |
| ***Stk17b*** | serine/threonine kinase 17b (apoptosis-inducing) | 0.00478115 | -1.55397 | 0.388221 | -1.09865 | 0.00215428 | -1.68041 | 0.00788654 | -1.48522 | 0.382957 | -1.09988 |
| ***Cxcr7*** | chemokine (C-X-C motif) receptor 7 | 0.00069296 | -1.55693 | 0.361327 | -1.07088 | 0.101458 | -1.14335 | 0.940304 | 1.00543 | 0.190793 | 1.1076 |
| ***Acta2*** | actin, alpha 2, smooth muscle, aorta | 0.00040239 | -1.56115 | 0.00016743 | -1.68586 | 0.556627 | 1.04001 | 0.0294884 | 1.1962 | 0.0447237 | -1.17285 |
| ***Casp1*** | caspase 1 | 0.00550421 | -1.57109 | 0.945496 | 1.00764 | 0.145072 | -1.19585 | 0.170953 | -1.18069 | 0.607445 | -1.05959 |
| ***Pdgfrb*** | platelet derived growth factor receptor, beta polypeptide | 0.00054583 | -1.58468 | 0.983341 | 1.0015 | 0.623162 | -1.03633 | 0.400466 | -1.06435 | 0.118542 | -1.13371 |
| ***Irf2*** | interferon regulatory factor 2 | 0.00175485 | -1.5983 | 0.00042632 | -1.84692 | 0.0012617 | -1.64886 | 0.00010551 | -2.20222 | 0.0984506 | 1.1871 |
| ***Cd99*** | CD99 antigen | 0.00772038 | -1.59851 | 0.00140551 | -1.94693 | 0.00554196 | -1.65561 | 0.00340319 | -1.74799 | 0.04843 | 1.34303 |
| ***Gpx8*** | glutathione peroxidase 8 (putative) | 0.00076558 | -1.60075 | 0.0630507 | -1.18637 | 0.0131418 | -1.29847 | 0.0026528 | -1.44682 | 0.818816 | 1.01812 |
| ***Mmp23*** | matrix metallopeptidase 23 | 0.00196946 | -1.6009 | 0.319346 | -1.10274 | 0.154897 | -1.15784 | 0.184783 | -1.14448 | 0.537944 | 1.06059 |
| ***Ifngr1*** | interferon gamma receptor 1 | 0.00465318 | -1.60212 | 0.105013 | -1.22773 | 0.00530592 | -1.58152 | 0.0011197 | -1.87211 | 0.018417 | 1.41193 |
| ***Timp3*** | tissue inhibitor of metalloproteinase 3 | 0.00080712 | -1.62766 | 0.0142478 | -1.30731 | 0.656331 | -1.03741 | 0.00126335 | -1.5639 | 0.630007 | -1.04063 |
| ***Serpinb1a*** | serine (or cysteine) peptidase inhibitor, clade B, member 1a | 1.72E-05 | -1.63077 | 0.0429276 | -1.10667 | 0.127686 | -1.07247 | 0.0123883 | -1.14996 | 0.303562 | 1.04556 |
| ***Tgfbi*** | transforming growth factor, beta induced | 0.00342256 | -1.63957 | 0.0132693 | -1.44399 | 5.53E-05 | -2.90581 | 4.35E-05 | -3.04263 | 0.00062435 | 1.99217 |
| ***Cxcl16*** | chemokine (C-X-C motif) ligand 16 | 0.0140014 | -1.648 | 0.319437 | -1.17135 | 0.0167232 | -1.6139 | 0.0342361 | -1.48833 | 0.525613 | 1.10315 |
| ***Tnfaip8*** | tumor necrosis factor, alpha-induced protein 8 | 0.00095127 | -1.69029 | 0.825293 | 1.02032 | 0.00661938 | -1.42562 | 0.0761349 | -1.20531 | 0.514883 | 1.06223 |
| ***Tnfsf10*** | tumor necrosis factor (ligand) superfamily, member 10 | 0.00072721 | -1.7172 | 0.474651 | 1.0673 | 0.0171393 | -1.32169 | 0.0168899 | -1.32302 | 0.00161698 | 1.59022 |
| ***Bcl2l11*** | BCL2-like 11 (apoptosis facilitator) | 0.00017532 | -1.72126 | 0.126576 | -1.12432 | 0.0946836 | -1.14 | 0.191541 | -1.10214 | 0.0670135 | -1.159 |
| ***Dnajc5*** | DnaJ (Hsp40) homolog, subfamily C, member 5 | 0.0328078 | -1.74053 | 0.179268 | -1.35681 | 0.218549 | -1.31757 | 0.0115459 | -2.05436 | 0.0535339 | 1.61779 |
| ***Tgfb1*** | transforming growth factor, beta 1 | 0.00605274 | -1.76167 | 0.171223 | -1.23656 | 0.15749 | -1.2469 | 0.115137 | -1.28614 | 0.579653 | -1.08327 |
| ***Col4a5*** | collagen, type IV, alpha 5 | 0.00032557 | -1.76246 | 0.00407529 | -1.41576 | 0.15811 | -1.13241 | 0.140548 | -1.13991 | 0.0374265 | -1.22796 |
| ***Xaf1*** | XIAP associated factor 1 | 0.00056906 | -1.77502 | 0.209952 | -1.12925 | 0.00028945 | -1.91526 | 3.83E-05 | -2.53697 | 0.00165251 | 1.59711 |
| ***Tgfbi*** | transforming growth factor, beta induced | 8.02E-05 | -1.78563 | 0.00047919 | -1.52199 | 4.66E-06 | -2.5808 | 1.70E-06 | -3.08205 | 3.35E-05 | 1.9648 |
| ***Cxcr4*** | chemokine (C-X-C motif) receptor 4 | 2.21E-05 | -1.81902 | 0.950861 | 1.00326 | 0.00015286 | -1.53115 | 0.18271 | 1.07918 | 0.79565 | -1.01379 |
| ***Irf7*** | interferon regulatory factor 7 | 0.00575833 | -1.82639 | 0.691013 | -1.06184 | 0.00994271 | -1.70548 | 0.0035325 | -1.94949 | 0.259198 | 1.19624 |
| ***Cd36*** | CD36 antigen | 0.00019745 | -1.83934 | 0.628108 | 1.03942 | 0.0322753 | -1.23384 | 0.600521 | 1.04275 | 0.310062 | -1.08762 |
| ***Col5a1*** | collagen, type V, alpha 1 | 8.95E-05 | -1.86828 | 0.00023847 | -1.68982 | 0.0855739 | -1.14881 | 0.0472319 | -1.18289 | 0.00984793 | -1.2854 |
| ***Aldh4a1*** | aldehyde dehydrogenase 4 family, member A1 | 0.00298759 | -1.88873 | 0.01373 | -1.57756 | 0.0843475 | -1.31451 | 0.0213517 | -1.50557 | 0.643295 | -1.06663 |
| ***Cd38*** | CD38 antigen | 0.00017243 | -1.88902 | 0.0584525 | -1.19722 | 0.00022481 | -1.8338 | 0.00057775 | -1.6653 | 0.106348 | -1.15782 |
| ***Adhfe1*** | alcohol dehydrogenase, iron containing, 1 | 7.60E-05 | -1.90758 | 6.61E-05 | -1.93849 | 0.00014238 | -1.78217 | 8.78E-05 | -1.87683 | 0.0130536 | 1.26635 |
| ***Mgst3*** | microsomal glutathione S-transferase 3 | 0.00148824 | -1.93105 | 0.212885 | -1.18076 | 0.0118497 | -1.52978 | 0.319825 | -1.13803 | 0.0171927 | 1.47553 |
| ***Ccnd1*** | cyclin D1 | 0.005203 | -1.94601 | 0.157964 | -1.28508 | 0.00769402 | -1.84337 | 0.0960661 | -1.35899 | 0.0225285 | 1.6069 |
| ***Gja1*** | gap junction protein, alpha 1 | 0.00179295 | -1.98562 | 0.807531 | -1.03336 | 0.00384737 | -1.80022 | 0.824478 | 1.03032 | 0.480957 | 1.10167 |
| ***Cd84*** | CD84 antigen | 8.56E-05 | -2.00315 | 0.657727 | -1.03528 | 0.00185781 | -1.48178 | 0.00647725 | -1.35511 | 0.771407 | 1.02288 |
| ***Tgfbi*** | transforming growth factor, beta induced | 0.00011847 | -2.04604 | 0.078965 | -1.18732 | 5.73E-06 | -3.35682 | 1.36E-05 | -2.84115 | 0.00011242 | 2.05978 |
| ***Col1a1*** | collagen, type I, alpha 1 | 5.79E-07 | -2.05316 | 3.81E-05 | -1.4219 | 0.00492153 | -1.15227 | 0.00030095 | -1.27616 | 0.0966609 | 1.06651 |
| ***Col14a1*** | collagen, type XIV, alpha 1 | 0.00139974 | -2.14394 | 0.0383763 | -1.43464 | 0.00177834 | -2.07047 | 0.00060533 | -2.44618 | 0.0310649 | 1.46618 |
| ***Cd83*** | CD83 antigen | 0.00010841 | -2.15433 | 0.0704116 | -1.20721 | 0.195486 | -1.13299 | 0.0915591 | -1.18771 | 0.294851 | -1.10337 |
| ***Gpt2*** | glutamic pyruvate transaminase (alanine aminotransferase) 2 | 1.37E-05 | -2.17317 | 0.0210844 | -1.20618 | 0.00852677 | -1.26151 | 0.00189106 | -1.37478 | 0.870752 | -1.01032 |
| ***Pdgfra*** | platelet derived growth factor receptor, alpha polypeptide | 2.52E-05 | -2.20609 | 0.00184062 | -1.4368 | 1.89E-06 | -3.42892 | 1.27E-06 | -3.7342 | 0.00018055 | 1.74994 |
| ***Ccng1*** | cyclin G1 | 0.00061993 | -2.23071 | 0.0911358 | -1.28054 | 0.00422824 | -1.7335 | 0.0104683 | -1.57041 | 0.0956468 | 1.27506 |
| ***Aif1*** | allograft inflammatory factor 1 | 6.36E-06 | -2.24415 | 0.0114116 | -1.21959 | 0.00094276 | -1.39459 | 0.00015539 | -1.58953 | 0.523709 | -1.03807 |
| ***Igfbp7*** | insulin-like growth factor binding protein 7 | 1.56E-06 | -2.27856 | 1.92E-05 | -1.71063 | 1.20E-06 | -2.36485 | 5.67E-07 | -2.65695 | 0.024776 | 1.14087 |
| ***Il10rb*** | interleukin 10 receptor, beta | 0.00066216 | -2.29338 | 0.0689629 | -1.32975 | 0.00134688 | -2.06479 | 0.00123379 | -2.09055 | 0.152215 | 1.23522 |
| ***Dpt*** | dermatopontin | 0.00013561 | -2.32704 | 2.87E-05 | -3.03394 | 0.00011982 | -2.3713 | 8.49E-05 | -2.50361 | 0.00269765 | 1.61851 |
| ***Bcl2a1a /// Bcl2a1b /// Bcl2a1d*** | B cell leukemia/lymphoma 2 related protein A1a /// B cell leukemia/lymphoma 2 related p | 9.57E-05 | -2.35572 | 0.0195862 | -1.34414 | 0.00015086 | -2.20398 | 0.00070382 | -1.81525 | 0.320051 | 1.10679 |
| ***Ccl6*** | chemokine (C-C motif) ligand 6 | 0.00013287 | -2.37515 | 0.0845222 | -1.22977 | 3.65E-05 | -2.96283 | 0.00216296 | -1.67138 | 0.092526 | 1.22175 |
| ***Klf6*** | Kruppel-like factor 6 | 9.39E-05 | -2.39851 | 0.0744772 | -1.22798 | 0.0159073 | -1.37259 | 0.801252 | 1.02538 | 0.344926 | -1.10255 |
| ***Cd44*** | CD44 antigen | 0.00029293 | -2.41962 | 0.697463 | -1.04931 | 0.0137886 | -1.50076 | 0.0828912 | -1.27795 | 0.00340264 | -1.73666 |
| ***Cd5l*** | CD5 antigen-like | 2.35E-07 | -2.43115 | 0.00033682 | -1.28858 | 1.88E-06 | -1.87015 | 6.64E-06 | -1.65693 | 5.23E-05 | -1.42414 |
| ***Col5a1*** | collagen, type V, alpha 1 | 2.20E-05 | -2.45773 | 9.91E-05 | -1.99577 | 0.0008289 | -1.59848 | 2.82E-05 | -2.36563 | 0.125165 | 1.14488 |
| ***Tlr1*** | toll-like receptor 1 | 0.00024221 | -2.50349 | 0.375765 | -1.11989 | 0.686147 | -1.05151 | 0.277204 | -1.15195 | 0.167927 | -1.20396 |
| ***Gpx7*** | glutathione peroxidase 7 | 0.00060662 | -2.56713 | 0.00125902 | -2.27228 | 0.0106725 | -1.69194 | 0.0752934 | -1.36244 | 0.794406 | -1.04001 |
| ***Prc1*** | protein regulator of cytokinesis 1 | 6.33E-05 | -2.59762 | 0.0125357 | -1.40656 | 0.00401875 | -1.54988 | 0.83409 | -1.02143 | 0.480253 | -1.07567 |
| ***Ccl6*** | chemokine (C-C motif) ligand 6 | 5.70E-05 | -2.59936 | 0.418796 | -1.08619 | 9.74E-06 | -3.65825 | 0.00017671 | -2.18496 | 0.480213 | 1.07431 |
| ***Aldh3a2*** | aldehyde dehydrogenase family 3, subfamily A2 | 1.73E-06 | -2.6151 | 0.00219951 | -1.30851 | 0.113476 | 1.10236 | 6.13E-06 | -2.17157 | 0.0459066 | -1.14119 |
| ***Ccr5*** | chemokine (C-C motif) receptor 5 | 0.00582995 | -2.69073 | 0.867603 | -1.04208 | 0.00483882 | -2.80096 | 0.0828403 | -1.6366 | 0.666774 | 1.11319 |
| ***G0s2*** | G0/G1 switch gene 2 | 7.67E-06 | -2.70476 | 4.91E-06 | -2.92664 | 0.082906 | -1.15698 | 6.35E-05 | -1.99411 | 0.461351 | -1.05674 |
| ***Igfbp3*** | insulin-like growth factor binding protein 3 | 1.75E-05 | -2.82054 | 0.00124775 | -1.61764 | 7.66E-06 | -3.30335 | 2.64E-06 | -4.19144 | 6.39E-05 | 2.28813 |
| ***Ccna2*** | cyclin A2 | 0.00013052 | -2.82129 | 0.00041396 | -2.32022 | 0.00223989 | -1.83976 | 0.589945 | -1.07052 | 0.382818 | -1.11932 |
| ***Serpina12*** | serine (or cysteine) peptidase inhibitor, clade A (alpha-1 antiproteinase, antitrypsin) | 8.17E-07 | -2.88879 | 9.88E-08 | -4.54051 | 7.73E-06 | 2.06356 | 0.00155406 | -1.32295 | 2.88E-08 | -6.42777 |
| ***Cd48*** | CD48 antigen | 0.0001098 | -2.89403 | 0.226207 | -1.17398 | 0.00034233 | -2.37709 | 0.0098 | -1.55746 | 0.240854 | -1.16744 |
| ***Ly86*** | lymphocyte antigen 86 | 6.89E-05 | -2.90771 | 0.00287549 | -1.70363 | 0.00068627 | -2.02202 | 0.00190566 | -1.7834 | 0.0444261 | -1.32161 |
| ***Cd74*** | CD74 antigen (invariant polypeptide of major histocompatibility complex, class II antig | 0.00026434 | -2.94859 | 0.0104256 | -1.68276 | 0.095114 | -1.3238 | 0.0075711 | -1.74934 | 0.0027489 | -1.99864 |
| ***Serpinb6a*** | serine (or cysteine) peptidase inhibitor, clade B, member 6a | 0.00026911 | -2.96314 | 0.456285 | -1.12043 | 0.0442302 | -1.4368 | 0.19145 | -1.23397 | 0.0954243 | -1.32626 |
| ***Cd93*** | CD93 antigen | 0.00075986 | -2.974 | 0.00655597 | -2.02811 | 0.0389528 | -1.5793 | 0.00249596 | -2.3751 | 0.675499 | -1.07934 |
| ***Ctgf*** | connective tissue growth factor | 0.00029578 | -2.99031 | 0.0219089 | 1.56831 | 0.00478201 | -1.89367 | 0.31568 | -1.17396 | 0.152209 | 1.27155 |
| ***Tgfbr2*** | transforming growth factor, beta receptor II | 3.59E-05 | -3.04397 | 0.00069479 | -1.92269 | 0.00022439 | -2.23634 | 0.00048912 | -2.00964 | 0.543486 | 1.06815 |
| ***Timp2*** | tissue inhibitor of metalloproteinase 2 | 0.00035641 | -3.06933 | 0.0137664 | -1.70616 | 0.0093897 | -1.79269 | 0.040808 | -1.49651 | 0.621163 | -1.08419 |
| ***Pdgfrb*** | platelet derived growth factor receptor, beta polypeptide | 0.00026894 | -3.05789 | 0.051782 | -1.4273 | 4.58E-05 | -4.62471 | 2.91E-05 | -5.24275 | 0.00031937 | 2.95438 |
| ***Cd53*** | CD53 antigen | 5.25E-07 | -3.45837 | 4.47E-05 | -1.7879 | 7.20E-06 | -2.21721 | 3.67E-06 | -2.44288 | 0.0336095 | -1.16451 |
| ***Col4a5*** | collagen, type IV, alpha 5 | 1.77E-05 | -3.4638 | 0.00019134 | -2.26524 | 0.00068135 | -1.91117 | 0.0147343 | -1.40826 | 0.52585 | -1.07043 |
| ***Col4a2*** | collagen, type IV, alpha 2 | 2.65E-06 | -3.5751 | 3.53E-05 | -2.26411 | 8.96E-05 | -2.00189 | 3.21E-05 | -2.29493 | 0.0556508 | -1.19424 |
| ***Cd52*** | CD52 antigen | 2.77E-05 | -3.7491 | 0.00064913 | -2.11959 | 0.0006774 | -2.10696 | 0.00046301 | -2.22548 | 0.0310132 | -1.38504 |
| ***Serpinh1*** | serine (or cysteine) peptidase inhibitor, clade H, member 1 | 2.27E-06 | -3.81847 | 3.00E-06 | -3.59121 | 2.64E-05 | -2.41184 | 3.84E-06 | -3.40721 | 0.18978 | -1.12023 |
| ***Cd44*** | CD44 antigen | 5.53E-05 | -3.86286 | 0.303779 | -1.16269 | 0.00080636 | -2.29817 | 0.00120176 | -2.16218 | 0.617711 | -1.07307 |
| ***Mmp12*** | matrix metallopeptidase 12 | 6.66E-07 | -3.89292 | 0.015965 | -1.23405 | 0.0020965 | -1.38624 | 0.0187682 | -1.224 | 0.185178 | -1.09936 |
| ***Col6a2*** | collagen, type VI, alpha 2 | 8.33E-05 | -3.94434 | 0.00505364 | -1.87769 | 0.00122202 | -2.31352 | 0.00177205 | -2.18192 | 0.382551 | 1.14774 |
| ***Cd93*** | CD93 antigen | 2.06E-05 | -4.56213 | 0.00030083 | -2.57305 | 0.0003311 | -2.53121 | 0.00045302 | -2.40297 | 0.270474 | -1.16638 |
| ***Col6a1*** | collagen, type VI, alpha 1 | 5.21E-06 | -4.64238 | 8.77E-05 | -2.56468 | 5.44E-05 | -2.78543 | 2.40E-05 | -3.25847 | 0.0239915 | 1.35526 |
| ***Aldh1b1*** | aldehyde dehydrogenase 1 family, member B1 | 2.25E-05 | -4.93791 | 1.55E-05 | -5.49224 | 2.47E-05 | -4.81282 | 0.00041223 | -2.59287 | 0.00292631 | 1.92211 |
| ***Col4a1*** | collagen, type IV, alpha 1 | 1.13E-05 | -4.98801 | 4.16E-05 | -3.60932 | 0.00010549 | -2.97331 | 1.55E-05 | -4.58868 | 0.317095 | 1.14128 |
| ***Cd36*** | CD36 antigen | 9.56E-07 | -5.03658 | 0.00056364 | -1.70127 | 3.22E-06 | -3.72892 | 5.56E-06 | -3.31831 | 0.00080936 | 1.64296 |
| ***Cdca3*** | cell division cycle associated 3 | 3.24E-06 | -5.07086 | 1.93E-05 | -3.30912 | 0.00073189 | -1.86794 | 0.0716644 | -1.24092 | 0.912687 | -1.01137 |
| ***Cdk1*** | cyclin-dependent kinase 1 | 8.35E-05 | -5.10176 | 0.00034738 | -3.53078 | 0.00131766 | -2.66987 | 0.0329979 | -1.61454 | 0.683847 | -1.07714 |
| ***Cd68*** | CD68 antigen | 2.00E-05 | -5.64216 | 0.111856 | -1.30708 | 0.00144532 | -2.22141 | 0.00084301 | -2.42329 | 0.189777 | -1.23687 |
| ***Col1a1*** | collagen, type I, alpha 1 | 2.93E-06 | -5.97679 | 0.0138131 | -1.44472 | 0.00155962 | -1.79504 | 0.00742359 | -1.52802 | 0.570374 | 1.0663 |
| ***Ccnb2*** | cyclin B2 | 2.52E-06 | -6.31564 | 0.00011014 | -2.61136 | 0.00304933 | -1.67258 | 0.943059 | 1.00804 | 0.45649 | -1.08932 |
| ***Col1a2*** | collagen, type I, alpha 2 | 3.06E-06 | -6.52735 | 2.13E-05 | -3.84223 | 5.48E-06 | -5.46997 | 7.97E-06 | -4.92335 | 0.0220432 | 1.41459 |
| ***Col6a3*** | collagen, type VI, alpha 3 | 1.38E-07 | -6.56424 | 7.90E-07 | -4.07012 | 1.76E-06 | -3.40904 | 8.14E-07 | -4.04174 | 0.547918 | 1.04376 |
| ***Cd36*** | CD36 antigen | 3.39E-05 | -7.12134 | 0.0253129 | -1.69713 | 7.51E-05 | -5.51649 | 0.00027148 | -3.88567 | 0.0613806 | 1.50762 |
| ***Stmn1*** | stathmin 1 | 3.34E-07 | -7.16038 | 1.15E-06 | -4.94921 | 1.20E-05 | -2.92365 | 0.00017067 | -1.96233 | 0.379475 | -1.08052 |
| ***Mmp2*** | matrix metallopeptidase 2 | 1.15E-08 | -7.8906 | 4.26E-08 | -5.25293 | 2.40E-07 | -3.46221 | 8.48E-08 | -4.38506 | 0.00401988 | -1.24619 |
| ***Cdc20*** | cell division cycle 20 | 0.00016485 | -8.1065 | 0.00042933 | -5.80468 | 0.00534603 | -2.92097 | 0.392161 | -1.26136 | 0.953277 | 1.0155 |
| ***Col1a2*** | collagen, type I, alpha 2 | 1.31E-06 | -8.34881 | 0.00022483 | -2.3887 | 9.55E-05 | -2.75877 | 0.00077948 | -1.99841 | 0.844074 | -1.02302 |
| ***Vim*** | vimentin | 3.26E-08 | -9.50843 | 5.24E-06 | -2.60401 | 3.64E-08 | -9.12456 | 1.04E-07 | -6.39656 | 1.74E-05 | 2.17961 |
| ***Tmsb10*** | thymosin, beta 10 | 2.03E-05 | -10.4051 | 0.00040496 | -3.96479 | 0.00030621 | -4.26231 | 8.85E-05 | -6.11833 | 0.110474 | 1.44083 |
| ***Gsta2*** | glutathione S-transferase, alpha 2 (Yc2) | 1.43E-06 | -12.0393 | 7.45E-06 | -6.54929 | 0.620255 | -1.07127 | 0.00417656 | -1.80602 | 0.00035421 | -2.59529 |
| ***Col3a1*** | collagen, type III, alpha 1 | 1.37E-08 | -12.1963 | 7.65E-08 | -6.52462 | 8.89E-09 | -14.7181 | 9.26E-09 | -14.4503 | 3.15E-06 | 2.72498 |
| ***Afp*** | alpha fetoprotein | 1.50E-06 | -40.0963 | 0.00210714 | -2.7639 | 0.107341 | -1.45241 | 0.344921 | -1.22406 | 0.456646 | 1.16989 |
| ***Adam23*** | a disintegrin and metallopeptidase domain 23 | 0.00228794 | 1.51706 | 0.0220911 | 1.28651 | 0.00061223 | -1.71153 | 0.889134 | -1.01203 | 0.00573422 | 1.41146 |
| ***Itga1*** | integrin alpha 1 | 0.0254201 | -1.53932 | 0.674979 | -1.0664 | 0.159506 | -1.26401 | 0.0729654 | -1.37274 | 0.0668236 | 1.38558 |
| ***Ilk*** | integrin linked kinase | 0.0253822 | -1.54214 | 0.149719 | -1.27371 | 0.0873014 | -1.34853 | 0.0183417 | -1.60063 | 0.600869 | 1.08423 |
| ***Itgb3bp*** | integrin beta 3 binding protein (beta3-endonexin) | 0.00152725 | -1.55205 | 0.405247 | 1.07428 | 0.356015 | -1.08332 | 0.389446 | 1.07707 | 0.7854 | 1.02306 |
| ***Adamts2*** | a disintegrin-like and metallopeptidase (reprolysin type) with thrombospondin type 1 mo | 0.00884436 | -1.56908 | 0.763702 | -1.03788 | 0.267561 | -1.15536 | 0.626219 | -1.06252 | 0.737838 | 1.04231 |
| ***Adam8*** | a disintegrin and metallopeptidase domain 8 | 0.00867619 | -1.58386 | 0.217088 | -1.18016 | 0.229833 | -1.17415 | 0.0423639 | -1.36159 | 0.405112 | -1.11354 |
| ***Adamts2*** | a disintegrin-like and metallopeptidase (reprolysin type) with thrombospondin type 1 mo | 0.00048767 | -1.62878 | 5.45E-05 | -2.06061 | 0.00345027 | -1.39609 | 0.00844577 | -1.31719 | 0.0380682 | -1.20865 |
| ***Itga6*** | integrin alpha 6 | 0.00091986 | -1.64951 | 0.280919 | -1.10289 | 7.16E-05 | -2.21743 | 0.0469369 | -1.22891 | 0.0857904 | 1.18504 |
| ***Itgal*** | integrin alpha L | 0.0128648 | -1.66187 | 0.0992819 | -1.32705 | 0.0293939 | -1.51149 | 0.06098 | -1.39687 | 0.555964 | -1.09476 |
| ***Itga4*** | integrin alpha 4 | 5.30E-05 | -1.72638 | 0.0509366 | -1.13975 | 0.00725483 | -1.2388 | 0.0159409 | -1.1956 | 0.169398 | -1.08757 |
| ***Itfg1*** | integrin alpha FG-GAP repeat containing 1 | 0.13287 | -1.73353 | 0.268265 | 1.47131 | 0.423683 | -1.31217 | 0.638497 | -1.16947 | 0.323021 | 1.40606 |
| ***Adamts12*** | a disintegrin-like and metallopeptidase (reprolysin type) with thrombospondin type 1 mo | 0.00014878 | -1.93039 | 0.0464346 | -1.21461 | 0.210224 | -1.1152 | 0.0442819 | -1.21793 | 0.599619 | -1.044 |
| ***Adam10*** | a disintegrin and metallopeptidase domain 10 | 0.0273053 | -1.93436 | 0.798727 | -1.06251 | 0.04426 | -1.78074 | 0.163436 | -1.43495 | 0.123957 | 1.50189 |
| ***Itgb2*** | integrin beta 2 | 2.84E-06 | -2.24667 | 0.0846085 | -1.10456 | 9.29E-06 | -1.93753 | 1.54E-05 | -1.83414 | 0.575536 | 1.02893 |
| ***Adamts5*** | a disintegrin-like and metallopeptidase (reprolysin type) with thrombospondin type 1 mo | 0.00410776 | -2.61852 | 0.122673 | -1.46845 | 0.0181471 | -1.99141 | 0.0991756 | -1.51745 | 0.638341 | 1.11167 |
| ***Itga8*** | integrin alpha 8 | 3.36E-05 | -6.69582 | 0.0832795 | -1.43177 | 0.00023742 | -3.84028 | 0.00011935 | -4.58227 | 0.223759 | 1.2644 |
| ***Cdh5*** | cadherin 5 | 0.0156238 | -1.6513 | 0.998786 | 1.00024 | 0.0793794 | -1.37287 | 0.124198 | -1.30784 | 0.845459 | -1.03104 |
| ***Ctnnd1*** | catenin (cadherin associated protein), delta 1 | 0.0179911 | -1.71676 | 0.376719 | -1.17336 | 0.608321 | -1.09476 | 0.0590412 | -1.47617 | 0.918267 | 1.01809 |
| ***Saa2*** | serum amyloid A 2 | 4.49E-09 | 15.7448 | 2.40E-07 | 4.12115 | 8.07E-08 | -5.47622 | 9.03E-07 | 3.10506 | 1.36E-07 | 4.7497 |
| ***Saa1*** | serum amyloid A 1 | 1.77E-08 | 11.5121 | 1.58E-06 | 3.15412 | 1.11E-06 | -3.38435 | 3.27E-06 | 2.76221 | 1.49E-06 | 3.19303 |
| ***Prtn3*** | proteinase 3 | 1.06E-06 | 13.8471 | 8.84E-07 | 15.039 | 1.26E-06 | -12.865 | 0.0170368 | 1.54191 | 1.38E-06 | 12.3685 |
| ***Serpina7*** | serine (or cysteine) peptidase inhibitor, clade A (alpha-1 antiproteinase, antitrypsin) | 2.50E-06 | 4.46937 | 1.07E-06 | 5.62889 | 0.214132 | -1.12878 | 0.00030738 | -1.91039 | 1.30E-06 | 5.32994 |
| ***Serpine2*** | serine (or cysteine) peptidase inhibitor, clade E, member 2 | 5.63E-06 | 4 | 4.63E-05 | 2.62268 | 1.70E-06 | 5.46941 | 4.17E-05 | 2.6692 | 0.00095985 | -1.74498 |
| ***Serpine1*** | serine (or cysteine) peptidase inhibitor, clade E, member 1 | 1.79E-06 | 3.74684 | 1.53E-06 | 3.88083 | 0.373787 | -1.07232 | 0.171089 | 1.1196 | 1.64E-06 | 3.81765 |
| ***Dnajc12*** | DnaJ (Hsp40) homolog, subfamily C, member 12 | 2.66E-06 | 11.9691 | 1.10E-05 | 7.01218 | 0.00094995 | -2.4089 | 0.00018856 | 3.27054 | 3.60E-05 | 4.89138 |
| ***Cxcl14*** | chemokine (C-X-C motif) ligand 14 | 1.57E-06 | 8.25131 | 2.63E-06 | 6.91868 | 4.65E-05 | -3.25621 | 0.407852 | 1.10638 | 1.02E-05 | 4.63886 |
| ***Cxcl1*** | chemokine (C-X-C motif) ligand 1 | 8.05E-07 | 5.49652 | 0.00628461 | -1.40047 | 1.23E-06 | -4.88348 | 0.00015076 | 1.9973 | 2.17E-06 | 4.22403 |
| ***Mt2*** | metallothionein 2 | 4.12E-08 | 8.19084 | 2.75E-07 | 4.61343 | 1.66E-06 | -3.09299 | 0.00017362 | 1.65736 | 6.99E-06 | 2.42216 |
| ***Mt1*** | metallothionein 1 | 1.45E-09 | 7.62984 | 1.32E-08 | 4.0821 | 9.53E-07 | -1.98515 | 4.31E-06 | 1.7006 | 4.33E-07 | 2.18861 |
| ***Orm2*** | orosomucoid 2 | 7.58E-08 | 7.9787 | 1.70E-08 | 14.4062 | 3.76E-08 | -10.3352 | 8.81E-05 | 1.86565 | 4.31E-08 | 9.80917 |
| ***Fgl1*** | fibrinogen-like protein 1 | 6.57E-09 | 5.87725 | 1.44E-06 | 2.04744 | 8.55E-06 | -1.69809 | 3.07E-06 | 1.87885 | 4.39E-06 | 1.81002 |
| ***Lcn2*** | lipocalin 2 | 1.39E-08 | 5.13216 | 2.02E-08 | 4.64843 | 2.44E-09 | -8.91618 | 0.563223 | -1.02465 | 2.75E-09 | 8.53518 |
| ***Qsox1*** | quiescin Q6 sulfhydryl oxidase 1 | 9.80E-05 | 5.11225 | 0.0262472 | 1.69003 | 0.013083 | -1.86575 | 0.357781 | -1.19516 | 0.00086023 | 2.99743 |
| ***Socs2*** | suppressor of cytokine signaling 2 | 4.52E-05 | 4.6617 | 0.0001844 | 3.32178 | 0.595058 | 1.08624 | 0.00159742 | -2.2311 | 2.62E-05 | 5.43641 |
| ***Hspa8*** | heat shock protein 8 | 1.62E-05 | 4.64173 | 2.26E-06 | 8.56707 | 0.00996659 | 1.57818 | 0.0175717 | 1.49046 | 5.97E-05 | 3.39783 |
| ***Ang*** | angiogenin, ribonuclease, RNase A family, 5 | 9.07E-08 | 4.50298 | 2.38E-06 | 2.38143 | 0.00183776 | -1.30403 | 0.330615 | 1.05449 | 3.05E-05 | 1.75194 |
| ***Aldh2*** | aldehyde dehydrogenase 2, mitochondrial | 0.00061914 | 4.35277 | 0.00136972 | 3.54159 | 0.0271048 | 1.92572 | 0.00258565 | 3.05068 | 0.260593 | 1.32315 |
| ***Malat1*** | metastasis associated lung adenocarcinoma transcript 1 (non-coding RNA) | 0.00084942 | 4.3519 | 0.443479 | 1.2168 | 0.135947 | 1.50964 | 0.175523 | 1.444 | 0.370759 | 1.2604 |
| ***Lrg1*** | leucine-rich alpha-2-glycoprotein 1 | 5.36E-06 | 4.19817 | 0.00048445 | 1.91477 | 0.00202063 | -1.63954 | 0.00319194 | 1.56976 | 0.00074856 | 1.8201 |
| ***Rb1cc1*** | RB1-inducible coiled-coil 1 | 0.00096931 | 3.73796 | 0.976869 | -1.00667 | 0.37 | 1.23754 | 0.686416 | 1.09773 | 0.584506 | 1.13549 |
| ***Igfbp1*** | insulin-like growth factor binding protein 1 | 2.51E-08 | 3.67597 | 2.87E-07 | 2.37581 | 5.92E-05 | -1.41681 | 0.00010237 | 1.37204 | 5.64E-07 | 2.16425 |
| ***Stat3*** | signal transducer and activator of transcription 3 | 2.38E-05 | 3.61657 | 0.923977 | -1.01101 | 0.921242 | -1.01141 | 0.0113707 | 1.48619 | 0.202374 | 1.1706 |
| ***Saa4*** | serum amyloid A 4 | 5.56E-07 | 3.47486 | 1.95E-05 | 1.97364 | 0.00410787 | 1.28813 | 0.00050444 | 1.46433 | 0.0052083 | 1.27232 |
| ***Prlr*** | prolactin receptor | 2.48E-06 | 3.43033 | 2.51E-06 | 3.42231 | 0.00017441 | -1.80383 | 0.00474607 | -1.3676 | 2.14E-06 | 3.53914 |
| ***Gas6*** | growth arrest specific 6 | 1.19E-05 | 3.40667 | 1.39E-05 | 3.29758 | 2.14E-06 | -5.1661 | 0.00358757 | -1.53881 | 4.15E-06 | 4.33744 |
| ***Rock1*** | Rho-associated coiled-coil containing protein kinase 1 | 0.00129419 | 3.38287 | 0.0665463 | 1.61759 | 0.399764 | 1.21501 | 0.929608 | -1.01999 | 0.226786 | 1.33559 |
| ***Rgs5*** | regulator of G-protein signaling 5 | 7.92E-05 | 3.34425 | 0.00039265 | 2.4729 | 0.0328682 | -1.42192 | 0.490227 | 1.09825 | 0.00054896 | 2.34196 |
| ***Os9*** | amplified in osteosarcoma | 0.0129337 | 3.29781 | 0.531129 | 1.25477 | 0.741857 | 1.12508 | 0.450563 | 1.31733 | 0.412853 | 1.35055 |
| ***Apcs*** | serum amyloid P-component | 1.19E-09 | 3.27545 | 5.33E-08 | 1.87411 | 4.58E-07 | -1.54919 | 0.00017415 | 1.17058 | 3.07E-08 | 1.99136 |
| ***Glul*** | glutamate-ammonia ligase (glutamine synthetase) | 4.25E-06 | 3.13184 | 2.46E-05 | 2.32774 | 2.86E-06 | 3.39178 | 0.012646 | 1.2912 | 0.00379983 | 1.39486 |
| ***Orm3*** | orosomucoid 3 | 6.64E-05 | 3.10692 | 8.20E-05 | 2.98106 | 3.72E-06 | -6.44583 | 0.969901 | 1.00458 | 3.73E-05 | 3.50554 |
| ***Serpina4-ps1*** | serine (or cysteine) peptidase inhibitor, clade A, member 4, pseudogene 1 | 3.15E-07 | 3.05925 | 0.00031468 | -1.40398 | 2.83E-06 | 2.16384 | 1.79E-08 | -6.09975 | 6.83E-05 | 1.56187 |
| ***Ctsc*** | cathepsin C | 0.0002005 | 2.8158 | 8.05E-05 | 3.38065 | 1.20E-05 | -5.45648 | 0.0483368 | -1.37578 | 6.03E-06 | 6.73662 |
| ***Pdap1*** | PDGFA associated protein 1 | 0.00040266 | 2.77752 | 0.260908 | 1.19655 | 0.543827 | 1.09747 | 0.0798278 | 1.35592 | 0.459379 | 1.12107 |
| ***Jak1*** | Janus kinase 1 | 0.0109244 | 2.70785 | 0.162546 | 1.54719 | 0.819743 | -1.06746 | 0.435524 | 1.25739 | 0.0892247 | 1.74256 |
| ***Gadd45g*** | growth arrest and DNA-damage-inducible 45 gamma | 7.32E-05 | 2.69448 | 0.00010386 | 2.53895 | 0.340165 | 1.11293 | 0.00026054 | -2.20365 | 0.0692576 | 1.25624 |
| ***Chka*** | choline kinase alpha | 0.00566713 | 2.68244 | 0.603222 | -1.1374 | 0.26593 | 1.33371 | 0.185894 | 1.41999 | 0.69421 | -1.10172 |
| ***Il6st*** | interleukin 6 signal transducer | 0.00041122 | 2.57148 | 0.0216862 | 1.51167 | 0.0861944 | -1.31674 | 0.784455 | -1.03913 | 0.00782367 | 1.6917 |
| ***Med1*** | mediator complex subunit 1 | 0.00011743 | 2.56137 | 0.149586 | -1.19249 | 0.0866666 | 1.24366 | 0.00215772 | -1.72734 | 0.731819 | -1.039 |
| ***Serpina10*** | serine (or cysteine) peptidase inhibitor, clade A (alpha-1 antiproteinase, antitrypsin) | 3.71E-08 | 2.56042 | 3.43E-06 | 1.55072 | 2.82E-06 | -1.57389 | 0.00247119 | -1.1441 | 3.14E-07 | 1.92895 |
| ***Egfr*** | epidermal growth factor receptor | 6.24E-05 | 2.76137 | 0.164047 | 1.17715 | 0.335988 | 1.1136 | 0.0366702 | 1.31716 | 0.391475 | 1.09966 |
| ***Hspb1*** | heat shock protein 1 | 3.62E-05 | 2.47688 | 0.0005997 | 1.73044 | 7.89E-06 | 3.25303 | 0.00431128 | 1.45088 | 0.00050123 | -1.76295 |
| ***Socs3*** | suppressor of cytokine signaling 3 | 0.00020203 | 2.40854 | 0.865346 | -1.01961 | 3.21E-05 | -3.37549 | 0.0134655 | -1.46185 | 0.0008001 | 1.97823 |
| ***Nktr*** | natural killer tumor recognition sequence | 0.00260426 | 2.39921 | 0.506428 | 1.13331 | 0.565228 | 1.11381 | 0.219976 | 1.27451 | 0.459642 | 1.15021 |
| ***Trat1*** | T cell receptor associated transmembrane adaptor 1 | 0.00235554 | 2.37891 | 0.00591225 | 2.0464 | 8.62E-05 | -4.96895 | 0.0257615 | 1.65937 | 0.15247 | 1.32536 |
| ***Fgf1*** | fibroblast growth factor 1 | 0.00027295 | 2.32961 | 0.0123722 | 1.48217 | 0.151081 | 1.20128 | 0.166173 | 1.19203 | 0.00710923 | 1.56216 |
| ***Ceacam1*** | carcinoembryonic antigen-related cell adhesion molecule 1 | 7.66E-05 | 2.28005 | 0.73634 | 1.03101 | 0.00590311 | 1.43428 | 0.79092 | -1.02429 | 0.99277 | -1.00082 |
| ***Osgin1*** | oxidative stress induced growth inhibitor 1 | 4.57E-06 | 2.2749 | 0.0759926 | -1.12031 | 2.51E-05 | -1.84723 | 7.21E-07 | -3.07695 | 4.77E-06 | 2.26172 |
| ***Irs2*** | insulin receptor substrate 2 | 1.23E-05 | 2.2133 | 0.00073812 | -1.46693 | 0.551936 | -1.03898 | 0.0278382 | -1.19148 | 0.058408 | 1.15214 |
| ***Pura*** | purine rich element binding protein A | 0.0118486 | 2.19688 | 0.985804 | -1.0041 | 0.68015 | 1.1003 | 0.481196 | -1.18024 | 0.437007 | 1.20173 |
| ***Ywhaz*** | tyrosine 3-monooxygenase/tryptophan 5-monooxygenase activation protein, zeta polypeptid | 0.0043342 | 2.19486 | 0.0113166 | 1.89035 | 0.107292 | 1.39708 | 0.0367383 | 1.60451 | 0.0961347 | 1.41677 |
| ***Ptpn11*** | protein tyrosine phosphatase, non-receptor type 11 | 0.00716791 | 2.18972 | 0.106699 | 1.45085 | 0.0997203 | 1.46481 | 0.0287227 | 1.75357 | 0.429565 | 1.18079 |
| ***Serpina3n*** | serine (or cysteine) peptidase inhibitor, clade A, member 3N | 8.11E-06 | 2.17716 | 1.84E-05 | 1.96627 | 0.0004915 | -1.45805 | 0.0374602 | 1.15878 | 1.11E-05 | 2.09085 |
| ***Saa3*** | serum amyloid A 3 | 0.00123465 | 2.08545 | 1.75E-05 | 4.86012 | 0.0315715 | -1.43097 | 0.00040881 | -2.47172 | 7.93E-05 | 3.37152 |
| ***Ldhd*** | lactate dehydrogenase D | 0.00032219 | 2.08177 | 0.00081414 | 1.85387 | 0.0629586 | 1.25477 | 0.0442398 | 1.28759 | 0.0257608 | 1.34104 |
| ***Garem*** | GRB2 associated, regulator of MAPK1 | 0.0165207 | 2.07242 | 0.0682234 | 1.63393 | 0.379885 | -1.23321 | 0.0710911 | 1.62316 | 0.431885 | 1.20481 |
| ***Serpina3k*** | serine (or cysteine) peptidase inhibitor, clade A, member 3K | 1.50E-05 | 2.06637 | 0.515689 | -1.04043 | 0.00135748 | 1.38057 | 0.00022618 | -1.56909 | 0.0836555 | 1.12629 |
| ***Nrp1*** | neuropilin 1 | 0.00045776 | 2.04028 | 0.101911 | 1.22068 | 0.63594 | -1.05286 | 0.314493 | -1.1201 | 0.00784081 | 1.49879 |
| ***Hmgcs1*** | 3-hydroxy-3-methylglutaryl-Coenzyme A synthase 1 | 4.86E-06 | 2.03645 | 3.89E-06 | 2.09342 | 0.0422411 | -1.12669 | 0.0164216 | 1.16537 | 1.75E-05 | 1.77051 |
| ***Dnajc1*** | DnaJ (Hsp40) homolog, subfamily C, member 1 | 0.0187529 | 1.98524 | 0.441769 | 1.1934 | 0.742014 | 1.07685 | 0.2604 | 1.30579 | 0.293196 | 1.28061 |
| ***Phlda1*** | pleckstrin homology-like domain, family A, member 1 | 7.29E-05 | 1.98062 | 0.297389 | 1.08458 | 0.00091041 | -1.53991 | 2.69E-06 | -3.34383 | 0.00012343 | 1.86386 |
| ***Hsp90ab1*** | heat shock protein 90 alpha (cytosolic), class B member 1 | 0.00157678 | 1.93667 | 0.171365 | -1.20698 | 0.0650016 | 1.31405 | 0.276214 | 1.15611 | 0.0365401 | -1.38346 |
| ***Hsp90aa1*** | heat shock protein 90, alpha (cytosolic), class A member 1 | 0.000277 | 1.87291 | 0.0463628 | -1.23069 | 0.0100372 | 1.35966 | 0.0592639 | 1.21241 | 0.00644188 | -1.40366 |
| ***Litaf*** | LPS-induced TN factor | 0.00010936 | 1.86323 | 0.00250689 | 1.41416 | 0.00013378 | -1.82282 | 0.0431542 | -1.19473 | 6.72E-05 | 1.97033 |
| ***Csnk2a1*** | casein kinase 2, alpha 1 polypeptide | 0.0136769 | 1.84754 | 0.215772 | -1.27936 | 0.517751 | 1.13012 | 0.0260267 | 1.68724 | 0.0497971 | -1.54688 |
| ***Fetub*** | fetuin beta | 4.01E-06 | 1.83748 | 0.00016514 | 1.37556 | 0.00012674 | -1.39697 | 0.208003 | 1.05565 | 7.24E-05 | 1.44654 |
| ***Bnip3*** | BCL2/adenovirus E1B interacting protein 3 | 2.09E-05 | 1.82512 | 0.00366612 | 1.26136 | 0.00024216 | 1.47808 | 0.306003 | -1.05801 | 0.00044857 | 1.41779 |
| ***Il13ra1*** | interleukin 13 receptor, alpha 1 | 0.00584782 | 1.78807 | 0.00557649 | 1.79876 | 0.00075682 | -2.39771 | 0.44622 | -1.12013 | 0.00048429 | 2.58719 |
| ***Dnajc3*** | DnaJ (Hsp40) homolog, subfamily C, member 3 | 0.00021563 | 1.77474 | 0.524435 | 1.05018 | 0.388444 | -1.06968 | 0.681741 | 1.0317 | 0.362211 | 1.07406 |
| ***Dap*** | death-associated protein | 0.00098971 | 1.76671 | 0.0500532 | 1.26259 | 0.123537 | -1.18612 | 0.912333 | -1.011 | 0.0293287 | 1.31166 |
| ***Serpina11*** | serine (or cysteine) peptidase inhibitor, clade A (alpha-1 antiproteinase, antitrypsin) | 2.90E-05 | 1.68848 | 0.781083 | 1.01359 | 0.00032913 | 1.4056 | 0.442277 | -1.03893 | 0.0313464 | 1.13863 |
| ***Il1r1*** | heat shock protein 1B | 0.00087237 | 1.63742 | 0.0159318 | 1.30734 | 0.00653871 | -1.38899 | 0.00320075 | 1.46517 | 0.026402 | -1.26615 |
| ***Hspa1b*** | interleukin 1 receptor, type I | 0.00013196 | 1.64188 | 0.0795799 | -1.12852 | 1.05E-07 | 5.37123 | 1.51E-08 | 10.2247 | 0.00165729 | -1.36335 |
| ***Il13ra1*** | interleukin 13 receptor, alpha 1 | 0.0039865 | 1.59163 | 0.00447406 | 1.57409 | 0.00038971 | -2.0744 | 0.721674 | 1.03908 | 0.00028326 | 2.16735 |
| ***Il17rb*** | interleukin 17 receptor B | 0.0022728 | 1.59033 | 0.00147695 | 1.65717 | 0.0248902 | -1.31207 | 0.0658428 | -1.22778 | 6.75E-05 | 2.43395 |
| ***Vegfa*** | vascular endothelial growth factor A | 0.0129507 | 1.58732 | 0.08451 | 1.31414 | 0.918294 | 1.01426 | 0.267674 | 1.17543 | 0.0635123 | 1.35065 |
| ***Mmp14*** | matrix metallopeptidase 14 (membrane-inserted) | 0.00179887 | 1.58138 | 0.444695 | -1.07303 | 6.62E-05 | -2.32052 | 0.00025135 | -1.94178 | 0.00050731 | 1.79222 |
| ***Bid*** | BH3 interacting domain death agonist | 0.00527559 | 1.54385 | 0.00418362 | 1.57785 | 0.658674 | 1.0484 | 0.0198766 | -1.37751 | 0.00362474 | 1.59977 |
| ***Cxcl12*** | chemokine (C-X-C motif) ligand 12 | 3.46E-05 | 1.52885 | 0.00167983 | 1.23254 | 0.00011706 | -1.4086 | 6.31E-05 | -1.4654 | 2.20E-06 | 1.97465 |
| ***Igf1*** | Insulin-like growth factor 1 | 0.00014513 | 1.32128 | 0.0179883 | -1.11155 | 2.54E-05 | -1.45984 | 1.10E-06 | -1.90872 | 4.08E-07 | 2.14732 |
